# Atypia detected during breast screening and subsequent development of cancer: observational analysis of the Sloane atypia prospective cohort in England

**DOI:** 10.1136/bmj-2023-077039

**Published:** 2024-02-01

**Authors:** Karoline Freeman, David Jenkinson, Karen Clements, Matthew G Wallis, Sarah E Pinder, Elena Provenzano, Hilary Stobart, Nigel Stallard, Olive Kearins, Nisha Sharma, Abeer Shaaban, Cliona Clare Kirwan, Bridget Hilton, Alastair M Thompson, Sian Taylor-Phillips

**Affiliations:** 1 Warwick Screening, Division of Health Sciences, Warwick Medical School, University of Warwick, Coventry, UK; 2 Screening Quality Assurance Service, NHS England, Birmingham, UK; 3 Cambridge Breast Unit and NIHR Cambridge Biomedical Research Centre, Cambridge University Hospitals NHS Trust, Cambridge, UK; 4 School of Cancer & Pharmaceutical Sciences, King's College London, London, UK; 5 Comprehensive Cancer Centre at Guy’s Hospital, King’s College London, London, UK; 6 Histopathology and NIHR Cambridge Biomedical Research Centre, Cambridge University Hospitals NHS Foundation Trust, Cambridge, UK; 7 Patient representative, Independent Cancer Patients’ Voice, UK; 8 Warwick Clinical Trials Unit, Warwick Medical School, University of Warwick, Coventry, UK; 9 Screening Quality Assurance Service, NHS England, Birmingham, UK; 10 Breast Screening Unit, Seacroft Hospital, York Road, Leeds, UK; 11 University Hospitals Birmingham NHS Foundation Trust, Queen Elizabeth Hospital Birmingham, Edgbaston, Birmingham, UK; 12 Division of Cancer Sciences, School of Medical Sciences, Faculty of Biology, Medicine and Health, University of Manchester, Manchester, UK; 13 Department of Surgical Oncology, Dan L Duncan Comprehensive Cancer Center, Baylor College of Medicine, Houston, TX, USA

## Abstract

**Objective:**

To explore how the number and type of breast cancers developed after screen detected atypia compare with the anticipated 11.3 cancers detected per 1000 women screened within one three year screening round in the United Kingdom.

**Design:**

Observational analysis of the Sloane atypia prospective cohort in England.

**Setting:**

Atypia diagnoses through the English NHS breast screening programme reported to the Sloane cohort study. This cohort is linked to the English Cancer Registry and the Mortality and Birth Information System for information on subsequent breast cancer and mortality.

**Participants:**

3238 women diagnosed as having epithelial atypia between 1 April 2003 and 30 June 2018.

**Main outcome measures:**

Number and type of invasive breast cancers detected at one, three, and six years after atypia diagnosis by atypia type, age, and year of diagnosis.

**Results:**

There was a fourfold increase in detection of atypia after the introduction of digital mammography between 2010 (n=119) and 2015 (n=502). During 19 088 person years of follow-up after atypia diagnosis (until December 2018), 141 women developed breast cancer. Cumulative incidence of cancer per 1000 women with atypia was 0.95 (95% confidence interval 0.28 to 2.69), 14.2 (10.3 to 19.1), and 45.0 (36.3 to 55.1) at one, three, and six years after atypia diagnosis, respectively. Women with atypia detected more recently have lower rates of subsequent cancers detected within three years (6.0 invasive cancers per 1000 women (95% confidence interval 3.1 to 10.9) in 2013-18 *v* 24.3 (13.7 to 40.1) in 2003-07, and 24.6 (14.9 to 38.3) in 2008-12). Grade, size, and nodal involvement of subsequent invasive cancers were similar to those of cancers detected in the general screening population, with equal numbers of ipsilateral and contralateral cancers.

**Conclusions:**

Many atypia could represent risk factors rather than precursors of invasive cancer requiring surgery in the short term. Women with atypia detected more recently have lower rates of subsequent cancers detected, which might be associated with changes to mammography and biopsy techniques identifying forms of atypia that are more likely to represent overdiagnosis. Annual mammography in the short term after atypia diagnosis might not be beneficial. More evidence is needed about longer term risks.

## Introduction

Breast screening programmes aim to identify malignancies early when treatment is more effective in reducing breast cancer mortality, but also cause overdiagnosis and overtreatment of cancer that would not have presented symptomatically within the person’s lifetime.^1^ In addition to breast cancer, breast screening programmes identify an increasing number of lesions of uncertain malignant potential (B3), including those with epithelial atypia. Follow-up of atypia might further contribute to overdiagnosis, therefore current management strategies are controversial.

Atypia refers to the histopathological diagnosis of cytological atypia with or without architectural aberration and is diagnosed in 5-10% of needle biopsies performed as part of the English breast screening programme.^2^ ^3^ However, the term atypia includes diverse abnormalities, including atypical ductal hyperplasia (ADH), flat epithelial atypia (FEA), and lobular neoplasia, which includes atypical lobular hyperplasia (ALH) and lobular carcinoma in situ (LCIS). These processes are not malignant themselves, however cancer can coexist with these lesions.^3^ ^4^ Additionally, the presence of atypia has been found to confer a fourfold increased long term risk of subsequent breast cancer over a median follow-up of 15.7 years in a meta-analysis of 13 studies, including a total of 1759 women.^5^ This meta-analysis synthesised mainly small studies (median 92 women) from 1987 to 2010, spanning changes to screening programmes, imaging technology, atypia definitions and treatment options, and reported pooled relative risks of cancer development for a range of follow-ups from 6.8 to 21 years. Studies did not consider short term risk at three or six years (time periods reflecting NHS breast screening programme further routine screening rounds). While the overall increased risk is apparent, this is of limited use for policy makers in countries where routine screening is available. In particular, the important question is whether additional mammographic screens for such women are required to detect subsequent cancers earlier.

English guidelines recommend vacuum assisted excision for all atypias (except when associated with a papillary lesion, which requires assessment of the extent in continuity of the atypia) followed by annual mammographic surveillance.^6^ European consensus on the management of B3 lesions with atypia recommends excision by vacuum assisted biopsy of FEA and lobular neoplasia, followed by surveillance imaging for five years and open surgical excision for ADH.^7^ A second and third consensus in 2018 and 2023 stipulated that surveillance can only replace surgical excision of ADH in special situations after discussion at the multidisciplinary meeting.^8^ ^9^ In the United States, surgical excision is recommended for most ADH, for lobular neoplasia where imaging and pathology are discordant, and for FEA with ADH. For other atypias, surgical excision is not considered necessary and observation with clinical and imaging follow-up can be offered.^10^ Observation and follow-up are not further defined. The recommendations were based on evidence of upgrade rates to cancer on excision and long term cancer risk. However, evidence on the effectiveness of regular surveillance mammography was not available, which is of particular importance in countries where routine breast screening is not annual. Annual surveillance imaging is a safety net to ensure no cancers are missed at excision and provide an opportunity for early detection in women at high risk, but this approach is not evidence based. We need studies examining cancer detection over the short term, after a diagnosis of any type of atypia and after current diagnostic management. In England, for instance, annual surveillance is suggested after vacuum assisted excision of all forms of atypia, but with the provision that this should be amended when more data and national guidance become available.^11^

This study presents an analysis of the English Sloane Project prospective atypia cohort^12^ and reports the proportion of women with atypia who develop breast cancer by type of atypia and time frame. This evidence base will help policy makers to decide the requirements for surveillance mammography in the first five years after atypia detection.

## Methods

### Data sources

The Sloane Atypia Project comprises a prospective cohort of women diagnosed as having atypia through the NHS breast screening programme in the United Kingdom from April 2003 to the present. The dataset is formed from a prespecified prospective data collection form submitted to the Sloane Project that is based on preset standardised data collection expectations as part of national quality assurance processes. Centre level participation was voluntary, with processes implemented in recent years to provide participating centres with a list of eligible women to help participation and completeness.^12^ Data included information on women’s atypia type, age at diagnosis, mammographic features, biopsy method, histological features, surgical treatment, and adjuvant treatment up until June 2018 (supplementary method 1.1).

Data were matched by NHS number and date of birth at person level to the English Cancer Registry held by the National Cancer Registration and Analysis Service, and the Mortality and Birth Information System for information on subsequent development of breast cancer and mortality data until December 2018. Data were deidentified before sharing for analysis. Data collection, data cleaning, and verification methods are described in detail elsewhere.^13^ The present analysis followed our published protocol.^14^

### Inclusion criteria

**Fig 1:**
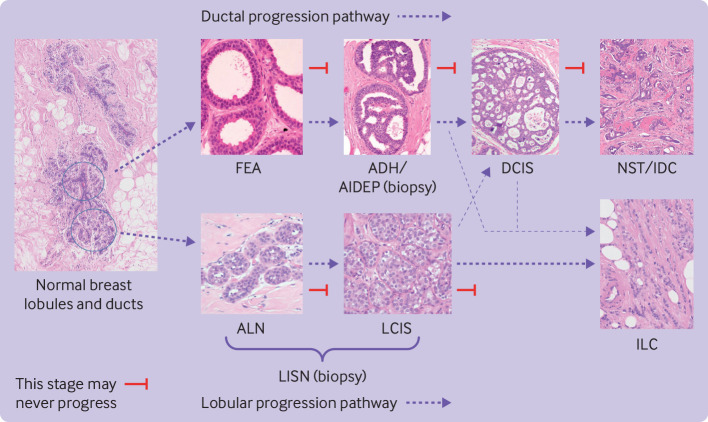
Overview depicting traditional views of association between different types of ductal and lobular atypia. Arrows describe potential ductal and lobular progression pathways. Thinner arrows represent rare progression of ductal precursors to ILC and lobular precursors to DCIS or invasive carcinoma of no special type (IDC). ADH=atypical ductal hyperplasia; AIDEP=atypical intraductal epithelial proliferation; ALH=atypical lobular hyperplasia; DCIS=ductal carcinoma in situ; FEA=flat epithelial atypia; IDC=invasive ductal carcinoma; ILC=invasive lobular carcinoma; LCIS=lobular carcinoma in situ; LISN=lobular in situ neoplasia; NST=no special type carcinoma

We identified all women on the Sloane database with epithelial atypia, and included those with ADH or atypical intraductal epithelial proliferation (AIDEP), FEA, ALH, and LCIS. Figure 1 depicts traditional views of the association between the different types of atypia to show how atypia types were considered in the analysis. We combined ALH, LCIS, and unspecified lobular in situ neoplasia (LISN) or LCIS under the term LISN. Supplementary method 1.2 defines types of atypia.

### Exclusion criteria

Women with ductal carcinoma in situ (DCIS) identified on the Sloane database were excluded; this group have been included in previous analyses.^15^ ^16^ ^17^ We excluded women with bilateral primary breast cancer when women had DCIS in one breast and atypia in the other, or the best prognosis atypia of the bilateral primaries in women with atypia in both breasts; patients with DCIS in addition to the atypia; those with pleomorphic LCIS (these are managed similarly to DCIS); those with an unknown type of atypia; women who were not from England; and women without linkage to the Mortality and Birth Information System to determine vital status on 31 December 2018.

### Follow-up

We followed women from six months after their atypia diagnosis until death (any cause) or 31 December 2018. For the primary analysis, follow-up was until the date of the first diagnosis of invasive breast cancer in either breast. For the secondary analysis, follow-up was until the date of the first diagnosis of DCIS or invasive breast cancer in either breast.

### Outcomes

The primary outcome was subsequent invasive breast cancer (see supplementary method 1.3 for information on collection and definition) per 1000 women diagnosed as having atypia at three years and six years after atypia diagnosis. This outcome was estimated from the cause specific cumulative incidence function calculated using time of observed cancer detection and time of death. Secondary outcomes included location of subsequent breast cancer, nature of subsequent cancer (grade, size, and nodal status), and cancers per 1000 women with atypia one year after their atypia diagnosis.

### Analysis

We summarised the characteristics of women with atypia, characteristics of atypia, and histological nature of subsequent cancer events for the whole cohort and by type of atypia using descriptive statistics. We recorded counts of breast cancer at one year, three years, and six years and investigated how diagnostic management changed over time. The number of deaths from breast cancer (see supplementary method 1.4 for definition) and the number of deaths from other causes were also reported.

For the primary analysis, we calculated cause specific cumulative incidence functions for invasive breast cancer (combined and split into ipsilateral and contralateral cancers) and death from any cause in a competing risks framework using the survfit function from the R package survival in R 4.1.2.^18^ The cumulative incidence function for invasive cancer was used to estimate the cumulative incidence of invasive cancers at one year, three years, and six years, with 95% confidence intervals. The three and six year time points represented the first and second rounds of screening after a diagnosis of atypia. The one year time point was a secondary analysis to explore missed cancers at the time of atypia diagnosis. We repeated the analysis for different types of atypia, age at atypia diagnosis, year of atypia diagnosis, and for different diagnostic management strategies to explore their effect on subsequent cancer rates. For the secondary analysis, we considered DCIS and invasive breast cancer as the outcome, with death as the competing risk at all three time points.

We undertook a sensitivity analysis including only consecutive women with atypia to explore the possibility of selective reporting of women with atypia to the Sloane Project, and a sensitivity analysis in which we excluded cancers detected within 12 months of atypia diagnosis as missed cancers. Supplementary method 1.5 reports the justification and approaches for all analyses. We reported the overall patterns of missing data by recording the number of unrecorded or missing items for each variable.

**Fig 2:**
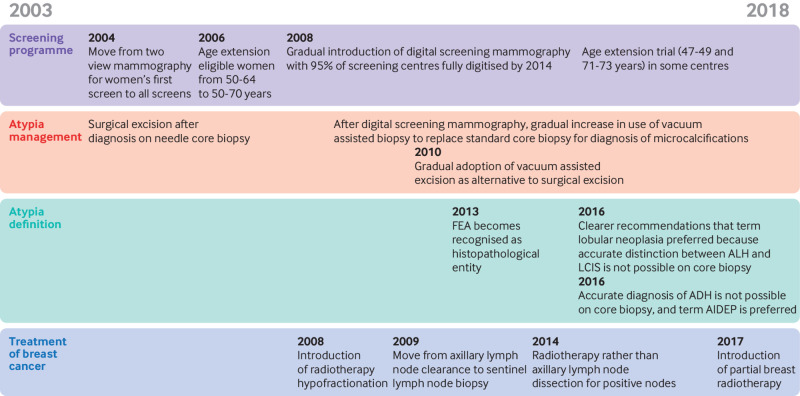
Major changes to screening and management of atypia during study period (2003-18). ADH=atypical ductal hyperplasia; AIDEP=atypical intraductal epithelial proliferation; ALH=atypical lobular hyperplasia; FEA=flat epithelial atypia; LCIS=lobular cancer in situ

We used flexible parametric models by following the method of Hinchliffe and Lambert^19^ to explore the effect of several explanatory variables on the time to breast cancer since atypia diagnosis using a competing risks framework and considering events as described above. This modelling approach produced hazard ratios with 95% confidence intervals. We considered age at diagnosis, year of diagnosis, type of atypia, management pathway, calcification, and background parenchymal breast density as explanatory variables, and consecutive versus non-consecutive women with atypia. Age was included as a continuous, linear variable (see supplementary method 1.6 for rationale). We calculated model fit statistics, Akaike’s information criterion, and Bayesian information criterion for model selection. A subdistribution model with the same covariates as the chosen model was also fitted (see supplementary method 1.7 for a discussion of both modelling approaches). Results were interpreted by considering major changes to the breast screening programme and the detection and management of atypia during the study period (fig 2).

### Patient and public involvement

Patients were involved in all stages of the project from grant application through to dissemination. Patients contributed to monthly project meetings, discussion of findings with patient groups, and to written reports and publicly available information.

## Results

### Characteristics of women and their atypia in Sloane cohort

A total of 3762 women with an atypia diagnosis after routine breast screening were reported to the Sloane Project in the UK between 1 April 2003 and 30 June 2018. Of these women, 3238 met our inclusion criteria (supplementary figure S1 and supplementary table S1). In total, women were reported from 63 of 77 (81.8%) English breast screening centres, however this proportion fluctuated over the study period. The mean age was 55.6 years (range 46-95) and the total follow-up was 19 087.9 person years. Of 3238 women with atypia, 1350 had ADH, 403 had FEA, 1101 had LISN, and 384 had mixed ductal and lobular atypia. Microcalcifications were present in 2525 (78%) diagnosed atypia.

**Fig 3:**
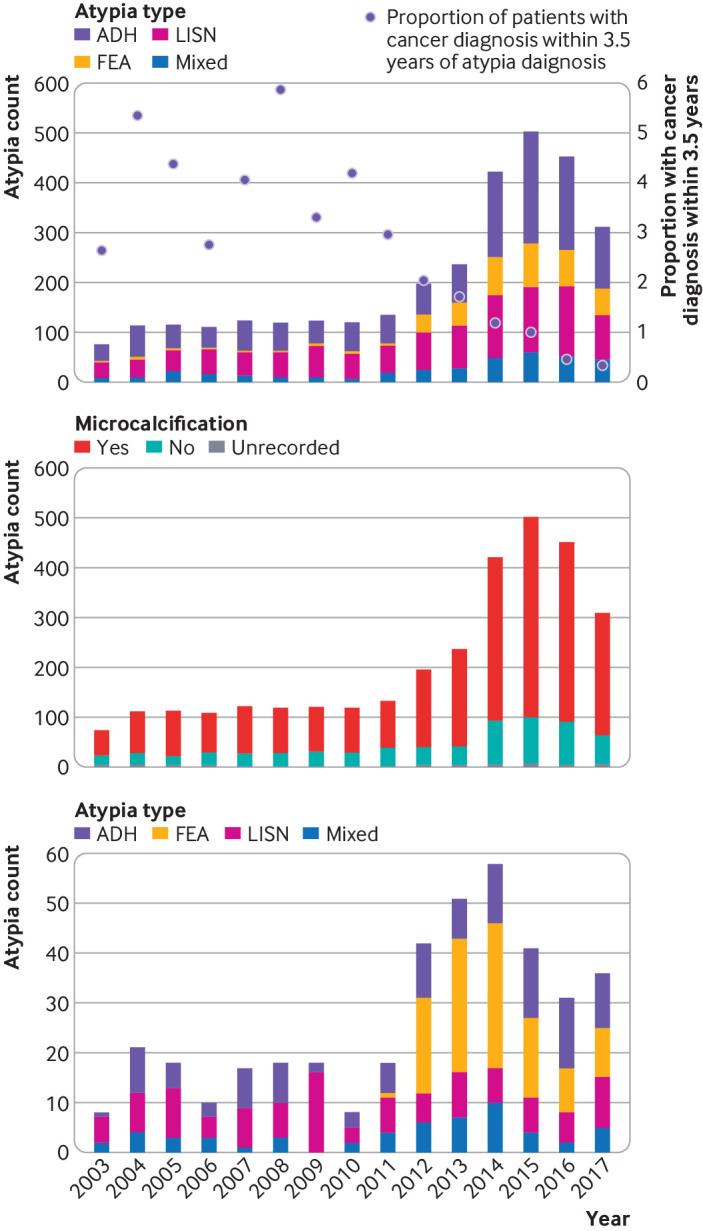
Number of atypia diagnoses by year. Upper panel: all centres by type of atypia, with proportion of women with invasive cancer diagnosis within 3.5 years of atypia diagnosis; middle panel: microcalcification present or absent; lower panel: two centres reporting consecutive women with atypia for complete study period. Transition from film screen to digital mammography occurred in 2010-13; FEA became recognised as histopathological entity in 2013. ADH=atypical ductal hyperplasia; FEA=flat epithelial atypia; LISN=lobular in situ neoplasia

There was a fourfold increase in the incidence of atypia between 2010 and 2015 (fig 3, upper panel). This increase cannot be explained by the 15% increase in women attending breast screening over the same time period^20^ or the change in age of women screened given the two age extensions during the study window. More women with atypia were recorded in the time period 2013-18 (n=2014) than in the previous two time periods (2003-07, n=534; 2008-12, n=690). This appeared to be a genuine increase in atypia numbers rather than an increase caused by more complete reporting because it was also apparent in centres that reported all women with atypia throughout the study period (fig 3, lower panel). While an increase in FEA diagnoses contributed to the overall increase, it was not the only reason (fig 3, top panel). FEA diagnoses increased over the three time periods in proportion to all atypia diagnoses (2.6% in 2003-07; 16.8% in 2013-18), while the relative numbers of the other atypia types showed minimal change but with an increase in absolute numbers (supplementary table S2). The increase in numbers of atypia coincided with an increase in the proportion of atypia with microcalcifications, which occurred around the time when digital mammography was introduced in screening centres between 2010 and 2013^21^ (fig 3, middle panel).

### Subsequent breast cancer events after atypia diagnosis

Of 3238 women with atypia with mean follow-up of 5.9 years (range 0.51-15.7), 168 (5.2%) developed breast cancer. Of these, 141 had invasive cancer and 27 had DCIS. Table 1 reports characteristics of invasive cancer for all atypia types and separately for each subtype. Supplementary table S3 presents characteristics of DCIS.

**Table 1:** Characteristics of subsequent invasive cancers (any time after original screening round and up until follow-up) detected after atypia diagnosis

Characteristics	All atypia	ADH or AIDEP	FEA	LISN	Mixed ductal and lobular
Women with atypia	3238	1350‡ (41.7)	403 (12.4)	1101 (34.0)	384 (11.9)
Women with subsequent breast cancer	168 (5.2)*	65 (4.8)	13 (3.2)	60 (5.4)*	30 (7.8)
Subsequent breast cancer: invasive	141† (83.9)	54 (83.1)	8 (61.5)	54 (90.0)	25† (83.3)
Site					
Ipsilateral cancer	82 (58.2)	29 (53.7)	4 (50.0)	32 (59.3)	17 (68.0)
Contralateral cancer	59 (41.8)	25 (46.3)	4 (50.0)	22 (40.7)	8 (32.0)
Grade					
1	25 (17.7)	9 (16.7)	1 (12.5)	8 (14.8)	7 (28.0)
2	69 (48.9)	27 (50.0)	5 (62.5)	28 (51.9)	9 (36.0)
3	28 (19.9)	15 (27.8)	1 (12.5)	7 (13.0)	5 (20.0)
Unrecorded	19 (13.5)	3 (5.6)	1 (12.5)	11 (20.4)	4 (16.0)
Size (mm)					
Median (interquartile range)	15.0 (9.75-27.25)	15.0 (10.0-24.75)	14.0 (13.25-14.75)	18.0 (10.0-30.0)	12.0 (8.5-22.0)
≤20	77 (54.6)	35 (64.8)	5 (62.5)	23 (42.6)	14 (56.0)
>20 to ≤50	32 (22.7)	14 (25.9)	0	13 (24.1)	5 (20.0)
>50	7 (5.0)	1 (1.9)	1 (12.5)	5 (9.3)	0
Unrecorded	25 (17.7)	4 (7.4)	2 (25.0)	13 (24.1)	6 (24.0)
Nodal status					
0 nodes positive	84 (59.6)	33 (61.1)	5 (62.5)	30 (55.6)	16 (64.0)
1, 2, or 3 nodes positive	22 (15.6)	12 (22.2)	0	6 (11.1)	4 (16.0)
>3 nodes positive	7 (5.0)	3 (5.6)	2 (25.0)	2 (3.7)	0
Unrecorded	28 (19.9)	6 (11.1)	1 (12.5)	16 (29.6)	5 (20.0)
Hormone receptor status					
Oestrogen receptor positive	108 (76.6)	39 (72.2)	7 (87.5)	44 (81.5)	18 (72.0)
Oestrogen receptor negative	10 (7.1)	8 (14.8)	0	1 (1.9)	1 (4.0)
Oestrogen receptor not known or unrecorded	23 (16.3)	7 (13.0)	1 (12.5)	9 (16.7)	6 (24.0)
Progesterone receptor positive	47 (33.3)	14 (25.9)	2 (25.0)	21 (38.9)	10 (40.0)
Progesterone receptor negative	10 (7.1)	5 (9.3)	0	5 (9.3)	0
Progesterone receptor not known or unrecorded	84 (59.6)	35 (64.8)	6 (75.0)	28 (51.9)	15 (60.0)
HER2 positive	15 (10.6)	5 (9.3)	0	5 (9.3)	5 (20.0)
HER2 negative	89 (63.1)	35 (64.8)	5 (62.5)	36 (66.7)	13 (52.0)
HER2 not known or unrecorded	37 (26.2)	14 (25.9)	3 (37.5)	13 (24.1)	7 (28.0)
Lymphovascular invasion					
Present	12 (8.5)	8 (14.8)	0	3 (5.6)	1 (4.0)
Possible	3 (2.1)	1 (1.9)	0	2 (3.7)	0
Absent	66 (46.8)	29 (53.7)	5 (62.5)	23 (42.6)	9 (36.0)
Not known or unrecorded	60 (42.6)	16 (29.6)	3 (37.5)	26 (48.1)	15 (60.0)

Data are numbers (%) unless indicated otherwise.

ADH=atypical ductal hyperplasia; AIDEP=atypical intraductal epithelial proliferation; FEA=flat epithelial atypia; HER2=human epidermal growth factor receptor 2; LISN=lobular in situ neoplasia.

* An additional two women had recorded distant metastasis, but no breast cancer recorded.

† This includes one woman with an invasive cancer recorded but no date of detection, who is therefore not included in the analysis of cancer rates at one, three, and six years after atypia diagnosis.

‡ This includes 326 (10.1%) women who received an AIDEP diagnosis.

The characteristics of the subsequent invasive cancers were similar to those of cancers detected in the general screening population. Most of the invasive cancers recorded were ≤20 mm in size and were node negative. The distribution of grades of the 141 invasive cancers detected was similar to screen detected cancers in the literature (see supplementary table S4): 25 (17.7%) grade 1, 69 (48.9%) grade 2, 28 (19.9%) grade 3, and 19 (13.5%) unrecorded.

The numbers of ipsilateral and contralateral invasive cancers at three years were similar (ipsilateral: 7.7 per 1000 women, 95% confidence interval 4.98 to 11.5; contralateral: 6.5, 3.99 to 10.1). While reporting was incomplete for the location of 22 ipsilateral cancers detected within three years of the initial atypia diagnosis (supplementary table S5), the number of contralateral cancers indicates that many atypia lesions are not direct precursors of subsequent breast cancers within the 15 years of follow-up available for analysis.

### Missed cancers at time of atypia diagnosis

The number of cancers diagnosed within 12 months probably reflects missed cancers at the time of the atypia diagnosis rather than cancers that developed after screening. Within six and 12 months after an atypia diagnosis, three invasive cancers were detected in women with atypia—one contralateral cancer after an ADH diagnosis and two ipsilateral cancers after a mixed atypia diagnosis. These findings equate to 0.95 (95% confidence interval 0.28 to 2.69) invasive cancers per 1000 women with atypia.

The main driver for an intensive follow-up of atypia is clinician concern about missing a cancer diagnosis when management of atypia moved from diagnostic surgical excision to vacuum assisted excision as a consequence of possible lower volume tissue removal. In the Sloane atypia cohort, the final atypia diagnosis was based on a single diagnostic procedure (standard core biopsy or vacuum assisted biopsy) in 477 (14.7%) women, a second line vacuum assisted biopsy or vacuum assisted excision in 964 (29.8%) women, and a surgical procedure in 1797 (55.5%) women. However, management with diagnostic surgical excision decreased and second line vacuum assisted excision increased during the study period (supplementary figure S3) in accordance with UK guidelines.^6^ This change in management strategy had little impact on numbers of invasive cancers detected. Second line vacuum assisted biopsy or vacuum assisted excision did not result in more cancers missed than surgery at one year (1.08 (0.11 to 5.9) *v* 1.12 (0.24 to 3.9) cancers per 1000 women, respectively) or three years (9.23 (4.1 to 18.4) *v* 18.5 (12.8 to 25.8) cancers per 1000 women, respectively). This finding applied to all atypia types and was independent of the site of cancer (supplementary table S6). The flexible parametric model confirmed that type of management had no effect (diagnostic surgical excision *v* second line vacuum assisted biopsy or vacuum assisted excision: hazard ratio 1.029, 95% confidence interval 0.54 to 1.95, P=0.93) when added after including age, year, and density; however, the wide confidence interval reflects the considerable uncertainty and means that a reduction in the hazard cannot be ruled out. Therefore, few cancers were missed at the time of atypia diagnosis and vacuum assisted excision appears to be as safe as surgical excision in the management of atypia.

### Cancers at three and six years after atypia and long term risk

**Fig 4:**
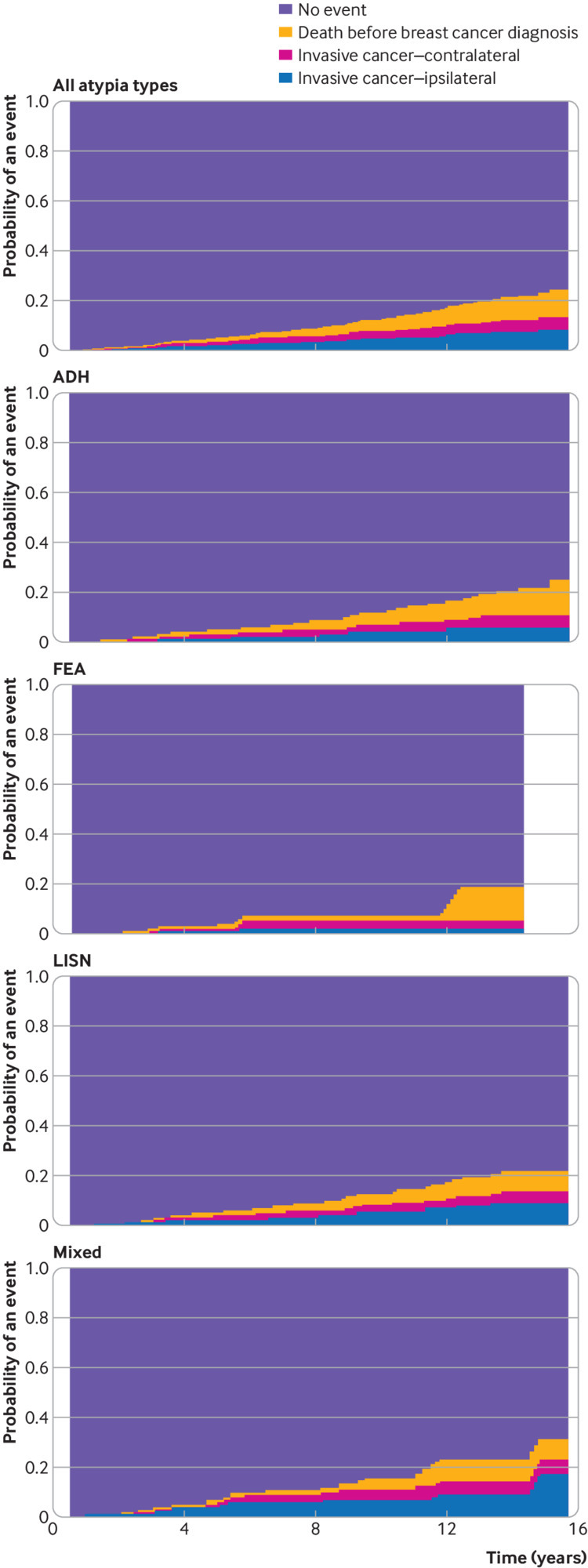
Cumulative incidence function for all atypia types and by atypia type for invasive cancer with death from any cause as competing risk. ADH=atypical ductal hyperplasia; FEA=flat epithelial atypia; LISN=lobular in situ neoplasia

The numbers of invasive cancers detected at three years and six years after an atypia diagnosis were estimated using the fitted cumulative incidence functions (three years: 14.2 per 1000 women, 95% confidence interval 10.3 to 19.1; six years: 45.0, 36.3 to 55.1; these figures are based on n=40 and n=94 invasive cancers detected, respectively; fig 4, table 2). While the number of cancers at three years was low, the number was higher at 3.5 years (23.8, 11.4 to 30.3), which presents a more pragmatic estimate because it includes cancers detected at the first routine (three yearly) screen after atypia when not all screens were on time. The numbers of cancers detected at three and six years after atypia diagnosis when an invasive cancer or DCIS was the outcome were estimated to be 18.9 per 1000 women (14.3 to 24.5; n=53) and 52.8 (43.4 to 63.4; n=113), respectively. Only one woman was reclassified in this analysis because she had a DCIS diagnosis followed by an invasive cancer.

**Table 2:** Cancers detected for complete study period and for three time periods expressed as counts and estimated from cumulative incidence function up to 1 year, 3 years, 3.5 years, and 6 years after atypia diagnosis

Calendar year at atypia diagnosis	No of atypia diagnoses	1 year			3 years			3.5 years			6 years	
		Absolute No of invasive cancers	Invasive cancers (95% CI)*		Absolute No of invasive cancers	Invasive cancers (95% CI)*		Absolute No of invasive cancers	Invasive cancers (95% CI)*		Absolute No of invasive cancers	Invasive cancers (95% CI)*
2003-18	3238	3	0.95 (0.28 to 2.69)		40	14.2 (10.3 to 19.1)		62	23.8 (11.4 to 30.3)		94	45.0 (36.3 to 55.1)
2003-07	534	0	0		13	24.3 (13.7 to 40.1)		21	39.3 (25.1 to 58.3)		36	67.4 (48.2 to 90.8)
2008-12	690	2	2.9 (0.61 to 9.94)		17	24.6 (14.9 to 38.3)		24	34.8 (22.9 to 50.4)		40	58.0 (42.2 to 77.1)
2013-18	2014	1	0.51 (0.055 to 2.89)		10	6.0 (3.09 to 10.9)		17	12.6 (7.5 to 20.0)		18	-

95% CI=95% confidence interval.

* Number per 1000 women.

Cancers by age at atypia diagnosis increased with age, apart from women aged 66-70 years (supplementary table S8). However, when considering age in combination with breast density and year of diagnosis in the flexible parametric model, neither age nor background parenchymal density had a clinically significant impact on cancer detection (supplementary figure S4). Furthermore, atypia type had no major impact on cancers detected (supplementary table S9). Adding atypia type as a variable to the model including age and year of diagnosis did not improve the model fit (supplementary table S13). Results from the models with cause specific hazards and subdistribution hazards gave the same conclusions (supplementary material 3). Therefore, there was no evidence that atypia management should be risk stratified by subgroup.

Fewer invasive cancers were detected at three years during 2013-18 (estimated to be 6.0 per 1000 women, 3.1 to 10.9) than during the two earlier time periods (2003-07: 24.3, 13.7 to 40.1; 2008-12: 24.6, 14.9 to 38.3) and remained low at 3.5 years (12.6, 7.5 to 20.0). These data suggest that the clinical significance of atypia diagnosed since 2013 was different from the effect of atypia diagnosed in earlier years. This finding was not caused by the lack of follow-up during the latest period (supplementary table S10) or the detection of more women with FEA in that time period. Excluding women with FEA from the analysis did not remove the observed difference (supplementary table S11). Furthermore, the reduced risk cannot be explained by selective reporting of more severe atypia diagnoses in the earlier time periods because the reduction in cancer rates was also substantial in an analysis of women with atypia from centres where all consecutive women with atypia were recorded (supplementary table S12). Additionally, the proportion of non-invasive to invasive breast cancers was higher in the latest time period than in previous time periods. Taken together, there were more atypia diagnoses and fewer cancers (but proportionally more DCIS) in the most recent time period (fig 3, top panel).

The cancer risk continued after six years (n=46 invasive cancers) in line with previous studies, with potentially slightly higher rates for mixed atypia and lowest rates for FEA at the end of follow-up (fig 4). However, care is needed in projecting long term risk from the earlier years to more recent atypia diagnoses, which could represent a different spectrum of atypia, and these lack the long term follow-up available for the earlier time periods.

### Mode of detection of subsequent breast cancers

Of 168 invasive breast cancers and DCIS, 57 (33.9%) were detected through screening and 47 (28.0%) were detected symptomatically; 32 (19.0%) cancers were detected by other outpatient appointments, which might have included annual screens. For 32 (19.0%) cancers, the mode of detection was not recorded. Supplementary figure S5 shows the mode of detection of subsequent cancers after an atypia diagnosis. Other outpatient appointments do not show an annual pattern, therefore we cannot assume cancers detected by other outpatient appointments were detected by annual surveillance mammography. A small number (12 of 168) of cancers were picked up symptomatically within the first three years after atypia diagnosis.

## Discussion

### Main findings

In the English Sloane atypia cohort of 3238 women with any epithelial atypia diagnosis, the incidence of atypia markedly increased from 2012 onwards. At the same time, detection of subsequent breast cancers in women with atypia decreased. Overall, cancer development after atypia was low compared with general population cancer rates and was considerably lower in more recent years than in earlier time periods. We propose that the gradual introduction of digital mammography in England since 2010, which identifies more microcalcifications,^22^ ^23^ could explain a large proportion of the increase in atypia from 2012 and might be the reason why lower rates of subsequent invasive cancers were detected in women with atypia from 2012 onwards. The remaining increase in atypia incidence might be because of a shift in atypia definitions and pathologists refining their diagnostic criteria, particularly the diagnosis and terminology of columnar cell lesions. FEA is one form of these lesions and appears to be uncommon before 2012. Another factor possibly relating to the increase in atypia could be the increased size of the biopsy needle that might have been used in recent years, increasing the probability of finding atypia and decreasing the probability of misclassifying atypia as DCIS.

Few cancers appeared to have been missed at the time of atypia diagnosis and non-surgical management was as safe as surgical excision of atypia in this cohort. The characteristics of cancers detected after atypia were similar to cancers detected in the general screening population and no subgroup was identified that was at increased risk of developing invasive cancer. Therefore, the reporting of atypia at screening could contribute to the problem of overdiagnosis in breast cancer screening.

### Comparison with previous studies

This study examines the short term risk of breast cancer after screen detected atypia. Previous studies^5^ ^24^ ^25^ ^26^ ^27^ ^28^ ^29^ ^30^ ^31^ ^32^ lack evidence to support a policy on the short term management of women after an atypia diagnosis because they focus on long term relative risks and only two studies have investigated atypia in a screening cohort. In Ireland, Boland and colleagues reported four cancers in 66 women with screen detected lobular neoplasia after mean follow-up of 62.5 months.^27^ Castells and colleagues reported on a cohort of women screened between 1994 and 2011 as part of the Spanish breast screening programme.^31^ In 159 women (0.029% of those screened), they recorded proliferative disease with atypia (although this included 28 benign or uncertain benign phyllodes tumours in this category, which is perhaps unexpected). Of these, six developed breast cancer (invasive or DCIS), which was equivalent to a cancer rate of 8.44 per 1000 person years compared with 7.7 per 1000 person years (9.2 considering invasive cancer and DCIS) in our study. In line with the results presented here, Castells and colleagues concluded that their results showed an association between benign breast disease and subsequent risk of cancer, with only a small number of malignancies misclassified as benign at biopsy and with no impact on cancer risk estimation. Considering all available follow-up (median 6.07 years), Castells and colleagues reported an age adjusted risk ratio of 4.56 (95% confidence interval 2.06 to 10.07) for women with atypia compared with women screened without benign disease (from first screen to cancer diagnosis), but with a similar pattern of time to breast cancer in both groups. However, the authors did not report estimates for the first five years after atypia diagnosis. Furthermore, none of the studies included women with atypia detected after 2011 when, according to our results, invasive cancers developed less frequently.

However, changes over time have been previously reported. An increase in lesions of uncertain malignant potential (B3 lesions), together with a decrease in the positive predictive value of malignancy for B3 lesions (in particular, lobular neoplasia) were reported in 2011 by Rakha and colleagues who compared B3 lesions detected in 1998-2000 with those detected in 2007-08.^33^ They reported a decrease in positive predictive value from 35% to 10% for B3 lesions, suggesting this decline was because of more accurate targeting of lobular neoplasia lesions by radiologists and more DCIS diagnoses with vacuum assisted biopsy (which would have been identified as AIDEP on the limited sampling provided by core biopsy).

### Strengths and limitations

The Sloane atypia prospective cohort comprises a large number of women compared with other predominantly retrospective studies or meta-analyses examining women with atypia and follow-up to cancer. However, the data have some limitations. Despite the substantial patient numbers, cancer after atypia diagnosis is rare, limiting the statistical power. Additionally, this is not a complete consecutive cohort across all English breast screening centres for the entire time period, so theoretically atypia lesions that are not included in the Sloane database (which is by voluntary submission) might be systematically different. Therefore, we compared our results for the whole cohort with those for the subset of centres known to have a complete, consecutive sample and they did not differ.

The cohort also encompasses a long time period, which enables assessment of temporal changes in the proportion of women who develop cancer. However, this long time period could complicate interpretation because several concurrent temporal changes play a part, such as improvement in imaging technology, changes in treatment and management of atypia, and changes in atypia terminology and definitions, and data collection forms. Furthermore, the data lacked information on symptomatic versus screen detected subsequent cancer and any data on annual surveillance mammography. Therefore, we gained little insight from the data about how atypia is currently managed, how subsequent cancers were detected, and which management strategy might work best in detecting cancers. Finally, the data lacked a comparator to assess cancer risk in a contemporary general screening population to put our findings into context.

### Implications for clinical practice

The results suggest that additional annual mammography for the first three years after a diagnosis of epithelial atypia might not be necessary over and above UK standard screening practice offered to all women (ie, once every three years). The number of women diagnosed as having atypia who developed cancer in the first three years was low. This cohort was not comparative, and so we cannot draw conclusions about the rate of cancers in women with atypia compared with the general screening population. However, the number of cancers detected within 3.5 years (one complete screening round per 1000 women with atypia) was 12.6 (95% confidence interval 7.5 to 20.0) in 2013-18. In the general population of women who have attended screening aged 50-70 years in 2018-19, the total rate of cancers within a three year screening round is comparable at 11.3 per 1000 women (3.5 symptomatically detected interval cancers between screening rounds^34^ and 7.8 detected at the next screening round^20^). Although without statistical comparisons or a matched cohort, these data suggest that the risk of developing cancer in the first 3.5 years is not high for women with atypia identified recently in a quality assured screening programme. The evidence is less clear for extra screening between three and five years when the rate of cancer is slightly higher than we would expect (58.0 per 1000 women, 95% confidence interval 42.2 to 77.1 at six years after atypia diagnosis). However, this evidence is for atypia diagnoses between 2008 and 2012 when digital mammography was not widely implemented and before the increase in numbers of atypia diagnoses, and with evidence for more recent years not yet available.

This study provides more limited data for longer term risks, although this was not the primary focus of the study. The National Institute for Health and Care Excellence states women in the general population have an 11% chance of developing breast cancer during their lifetime, with moderate risk greater than 17% but less than 30%.^35^ The 15 year risk in the Sloane cohort was 13.1% for the complete study period, however this estimate is less influenced by more recent atypia diagnoses that have shorter follow-up. However, 63 of 77 screening centres contributed data to the Sloane atypia cohort, which suggests that findings are applicable to screening practice generally in England. Other countries should interpret these findings with caution for policy decision making because of potential differences in breast image acquisition, access to vacuum assisted biopsies, the level of quality assurance of the screening programme, and the present management of atypia, which might increase the risk of overdiagnosis or overtreatment.

### Conclusion

Overall, data from the Sloane Atypia Project show that invasive breast cancer incidence at three years after a diagnosis of epithelial atypia was low, and even lower in recent years. Few cancers appeared to be missed at the time of an atypia diagnosis. These data, including the similar ipsilateral and contralateral risks, support the suggestion that many epithelial atypia diagnoses might represent risk factors rather than precursor lesions for invasive cancer within 15 years of follow-up. Changes to mammography (digital *v* plain film) and biopsy techniques (gauge of biopsy needle and use of vacuum assistance) coincide with the reduction in reported subsequent invasive cancers. One possible interpretation might be that, more recently, milder forms of atypia have been detected, which are more likely to represent overdiagnosis. Annual mammography in the short term after atypia diagnosis might not be beneficial and should be reviewed. Previous studies have shown increased longer term risk of developing cancer with some forms of epithelial atypia, but not all. Even for those lesions with established long term risk (eg, ADH, ALH, LCIS), the data indicate that these women would not benefit from enhanced short term surveillance.

## What is already known on this topic

Breast lesions of uncertain malignant potential with atypia might confer a three to fourfold increased long term risk of subsequent breast cancer
Guidelines in the United Kingdom, Europe, and America recommend excision of atypia by vacuum assisted biopsy or open surgery followed by surveillance imaging
Management with five years of annual surveillance imaging is not evidence based, and length, frequency, and appropriateness are controversial

## What this study adds

Breast cancer diagnosis within three years of atypia was low, particularly in more recent years (since 2012), and might contribute to increased overdiagnosis in breast cancer screening
More frequent mammography for five years after atypia diagnosis might not be beneficial in quality assured breast screening programmes with universal use of digital mammography and vacuum assisted excision of indeterminate lesions; such surveillance protocols should be reviewed
No evidence was found that surgical removal of atypia is required to prevent missed cancers; vacuum assisted excision appears to be as safe as surgical excision in managing atypia

## Data Availability

Data are available upon reasonable request. Access to the Sloane Project data from external parties is governed by application to the breast screening research advisory committee and Office for Data Release. Data will only be released by the Sloane Project to researchers under approval and in an anonymised or depersonalised format and under a data sharing agreement.

## Web extra

Extra material supplied by authors

Web appendix: Supplementary material

## Notes

Contributors: KF and STP drafted the manuscript. STP, KC, MGW, SEP, EP, HS, NSt, OK, NSh, AS, AMT, and CCK contributed to the study conceptualisation and design. KC, BH, and OK contributed to data collection. KF and DJ undertook the analysis. All authors commented on the draft, read and approved the final version. The corresponding author attests that all listed authors meet authorship criteria and that no others meeting the criteria have been omitted. STP acts as the guarantor.

The lead author affirms that the manuscript is an honest, accurate, and transparent account of the study being reported; that no important aspects of the study have been omitted; and that any discrepancies from the study as originally planned (and, if relevant, registered) have been explained.

Dissemination to participants and related patient and public communities: The results have already been presented to representatives of appropriate patient groups. After publication, results will be made available through Breast Cancer Now and to the broader public.

Provenance and peer review: Not commissioned; externally peer reviewed
