# Supplementary material for: Atypia detected during breast screening and subsequent development of cancer: observational analysis of the Sloane atypia prospective cohort in England
Source: BMJ. 2024 Feb 1;384:e077039. doi: 10.1136/bmj-2023-077039 (PMC10831586; doi:10.1136/bmj-2023-077039)

Supplementary material for Sloane atypia analysis

Content

1. ***Methods***
   1.1. Variables received from the Sloane Project data in accordance with the inclusion and exclusion criteria as per the Office for Data Release data sharing contract
   1.2. Atypia types
   1.3. Collection and definition of cancer events
   1.4. Definition of cause of death 'breast cancer'
   1.5. Additional analyses
   1.6. Options explored how to add age at diagnosis into the model
   1.7. Flexible parametric model choice – rationale

2. ***Results***

2.1 Study population

Figure S1 Flow diagram of study population

2.2 Characteristics of women and their atypia in the Sloane atypia cohort

Table S1 Descriptive statistics of all women with atypia for the study period April 2003 to June 2018
Table S2 Number of women with atypia and characteristics of subsequent cancers detected following atypia diagnosis by atypia type for three time periods separately
Figure S2 Proportion of atypia with microcalcifications present by year

2.3 Subsequent events following atypia

Table S3 Characteristics of subsequent invasive and non-invasive cancers by atypia type
Table S4 Distribution of grade for subsequent invasive cancers compared to published figures
Table S5 Location of 22 ipsilateral invasive cancers 3 years post atypia diagnosis by atypia type

2.4 Missed cancers at atypia diagnosis

Figure S3 Proportion of diagnostic management options performed by year
Table S6 Invasive cancers per 1000 women with atypia at 1 year and 3 years post atypia diagnosis by management strategy and site of invasive cancers separately for atypia types

2.5 Cancers at 3- and 6-years post atypia and long-term risk

Table S8 Invasive cancer rates per 1000 women with atypia at 1 year, 3 years and 6 years post atypia diagnosis by age group
Figure S4. Cumulative incidence function for invasive cancer and death from the main model, evaluated at 1 year, 3 years, and 6 years since diagnosis with atypia; by age at diagnosis, year of diagnosis and women's breast density (background parenchymal) (high figure 3a, low figure 3b).
Table S9 Invasive cancer rates at 3 years post atypia by atypia type
Table S10 Invasive cancer rates at 1 year, 3 years and 6 years post atypia diagnosis per 1000 women for 3 time periods under different scenarios
Table S11 Invasive cancer rates at 1 year, 3 years and 6 years post atypia diagnosis per 1000 women for 3 time periods excluding women with FEA

Table S12 Invasive cancer rates at 1 year, 3 years and 6 years post atypia diagnosis per 1000 women for 3 time periods for consecutive cases only

2.6 Mode of detection of subsequent cancers

Figure S5 Number of cancers (invasive and DCIS) over time since atypia diagnosis by mode of detection

3. Modelling

3.*1 Modelling of cancer rates using the cause specific hazard method*

Table S13 Model selection for models with causes of outcome: invasive cancer and death
Table S14 Cancer rates (fitted values) at 1, 3, and 6 years since atypia diagnosis from main model (by age, year of diagnosis and breast background parenchymal density). Cumulative incidence of invasive cancer per 1000 women
Table S15 Comparison of the risk of subsequent invasive cancers considering different factors
Figure S6 Cause-specific hazard function for each cause of outcome from the main model by time since atypia diagnosis.
Figure S7 Stacked cumulative incidence plots from the main model.
Figure S8 Cause-specific hazards (a, b), stacked cumulative incidence functions (c, d) and cumulative incidence functions (e, f) evaluated at 1 year, 3 years, and 6 years since diagnosis with atypia, from the main model with death and invasive cancer split into ipsilateral and contralateral.
Table S16 Fitted values at 1, 3, and 6 years since atypia diagnosis from main model with invasive cancer and DCIS combined.
Figure S9 Cumulative incidence functions evaluated at 1 year, 3 years, and 6 years since diagnosis with atypia, from the main model with invasive cancer or DCIS combined for high (a) and low (b) background parenchymal density

3.2 *Modelling of cancer rates using the subdistribution method*

Table S17 Cancer rates (fitted values) at 1, 3, and 6 years since atypia diagnosis from main model, using the subdistribution method. Cumulative incidence of invasive cancer per 1000 women
Figure S10 Cumulative incidence function for outcomes of both causes from the main model using the subdistribution method, evaluated at 1 year, 3 years, and 6 years since diagnosis with atypia; by age at diagnosis, year of diagnosis and background parenchymal density (high in figure a, low in figure b)

1. *METHODS*

1.1 Variables received from the Sloane Project data in accordance with the inclusion and exclusion criteria as per the Office for Data Release data sharing contract

Pseudonymised tumour ID
dtmDOD
bytCauseOfDeath
apptdate
intNoOfPreviousScreenings
dtmDateOfMammogram
bytBackgroundPattern
bytPredominantRadiologicalFeature
strLesionMicrocalcification
bytNottinghamDefn
strSizeOfLesionApplicable
dblSIZEOBLDistFromNipple
dblSIZEOBLLengthLesion
dblSIZEOBLDiamLesion
dblSIZECCDistFromNipple
dblSIZECCLengthLesion
dblSIZECCDiamLesion
dblMaxEstimatedLesionSize
bytAgeAtMammogram
bytOperationNumber
OpCount
Op1Date
Op1Procedure
Op1AxNodesTaken
Op1Sentinel
Op1ANS
Op1ANC
Op2Date
Op2Procedure
Op2AxNodesTaken
Op2Sentinel
Op2ANS
Op2ANC
Op3Date
Op3Procedure
Op3AxNodesTaken
Op3Sentinel
Op3ANS
Op3ANC
Op4Date

Op4Procedure
Op4AxNodesTaken
Op4Sentinel
Op4ANS
Op4ANC
Op5Date
Op5Procedure
Op5AxNodesTaken
Op5Sentinel
Op5ANS
Op5ANC
Op6Date
Op6Procedure
Op6AxNodesTaken
Op6Sentinel
Op6ANS
Op6ANC
ysnCoreBiopsy
dblCoreBiopsyWeight
ysnMamotone
dblMamotoneWeight
ysnOpenBiopsy
dblOpenBiopsyWeight
ysnTherapeuticExcision
dblTherapeuticExcisionWeight
ysnCavityShaves
dblCavityShavesWeight
ysnImmediateReExcision
dblImmediatereexcisionweight
ysnDelayedReExcision
dbldelayedreexcisionweight
ysnCompletionMastectomy
ysnMastectomy
ysnHISTADH
ysnHISTLISN
ysnSamplesADH
ysnSamplesLISN
intNodesNoExaminedAxilla
intNodesNoPositiveAxilla
intNodesNoExaminedSentinel
intNodesNoPositiveSentinel
intNodesNoExaminedOther
intNodesNoPositiveOther

bytOestrogenReceptorStatus
strOestrogenReceptorStatusCutOff
bytProgesteroneReceptorStatus
strProgesteroneReceptorStatusCutOff
bytHER2ReceptorStatus
strHER2ReceptorStatusCutOff
LngPathologist
ysnCore14GuageDCIS
ysnCore14GuageADH
ysnCore14GuageLCIS
ysnCore14GaugeALH
ysnCore14GaugeFEA
ysnCore14GaugePLCIS
ysnCore14GuageLISN
ysnCore14GaugeAIDEP
ysnCore8_11GuageDCIS
ysnCore8_11GuageADH
ysnCore8_11GuageLCIS
ysnCore8_11GaugeALH
ysnCore8_11GaugeFEA
ysnCore8_11GaugePLCIS
ysnCore8_11GaugeLISN
bytDCISCoreGrade
bytCoreCalcificationPresent
ysnDiseasePresentInSurgicalSpecimenADH
ysnDiseasePresentInSurgicalSpecimenALH
ysnDiseasePresentInSurgicalSpecimenFEA
ysnDiseasePresentInSurgicalSpecimenLCIS
ysnDiseasePresentInSurgicalSpecimenPLCIS
ysnDiseasePresentInSurgicalSpecimenNone
ysnNoSurgicalSpecimen
ysnDiagnosticBiopsy
ysnDiagnosticBiopsyADH
ysnDiagnosticBiopsyLCIS
ysnDiagnosticBiopsyALH
ysnDiagnosticBiopsyFEA
ysnDiagnosticBiopsyPLCIS
ysnDiagnosticBiopsyLISN
ysnDiagnosticBiopsyAIDEP
ysnTheraputicBiopsy
ysnTheraputicBiopsyADH
ysnTheraputicBiopsyLCIS
ysnTheraputicBiopsyALH

ysnTheraputicBiopsyFEA
ysnTheraputicBiopsyPLCIS
ysnTheraputicBiopsyLISN
ysnTheraputicBiopsyAIDEP
ysnAnotherProcessAtMarginALH
ysnAnotherProcessAtMarginFEA
ysnAnotherProcessAtMarginLCIS
ysnAnotherProcessAtMarginPLCIS
ysnAnotherProcessAtMarginADH
ysnAnotherProcessAtMarginLISN
strRadiotherapyExternalBeam
strRadiotherapyExternalBeamNO
ysnAdjvTherapyRadiotherapy
ysnAdjvTherapyOther
ysnAdjvTherapyHormone
ysnAdjvTherapyNoFurther
bytRecurrenceType
dtmRecurrence
ysnDetectFUMammogram
ysnClinicalExamineRoutineFU
ysnGPReferralOPDClinic
ysnOther
strOther
strOther (2)
bytSiteOfDisease
strOtherSiteOfDisease
ysnTypeInvasive
ysntypeNonInvasiveDCIS
ysntypeNonInvasiveLCIS_ALH
bytGradeInvasive
bytGradeDCIS
intSizeDCIS
intSizeInvasive
intSizeWholeTumour
intNodesNumberExamined
intNodesNumberPositive
bytVascularInvasion
bytOestrogenReceptorStatus
strOestrogenReceptorStatusCutOff
bytOestrogenReceptorStatusType
bytProgesteroneReceptorStatus
strProgesteroneReceptorStatusCutOff
bytProgesteroneReceptorStatusType

bytHER2ReceptorStatus
strHER2ReceptorStatusCutOff
bytHER2ReceptorStatusType
ysnProcFWLE
ysnProcMastectomy
ysnProcAxillaryNode
strOtherSurgicalProcedure
ysnRadiotherapy
ysnChemotherapy
ysnHormoneTherapy
bytTypeHormoneTherapy
strOtherHormoneTherapy

Colour code:

	Patient vital status
	Radiology/mammogram
	Surgical and axillary procedures
	Pathology - includes diagnostic/therapeutic pathology
	Adjuvant treatment (i.e. radiotherapy, endocrine therapy, none)
	Recurrence/Further event data

1.2 Atypia types

Atypical Ductal Lesions

For this study we combined AIDEP and ADH in the ADH group. This decision was based on:

1) AIDEP was introduced as a term on the Sloane data collection form in 2019 following the UK national guidelines that the specific entity of ADH should not be diagnosed on standard core biopsy or diagnostic VAB. Therefore, only a small number of cases is expected.

2) Most AIDEP cases that are not upgraded to ductal carcinoma in situ (DCIS) are regarded as ADH on excision.

Lobular Lesions

Lobular neoplasia (LN) and lobular in situ neoplasia (LISN) are interchangeable terms and encompass the spectrum of lobular lesions from atypical lobular hyperplasia (ALH) through to lobular carcinoma in situ (LCIS).

In the UK, guidance is not to record a specific diagnosis of ALH or LCIS on core biopsy or diagnostic VAB because insufficient amounts of material are received to make the distinction with accuracy. Instead, the broader diagnosis of LISN is preferred. This view is reflected in the Sloane pathology data collection form from 2016 onwards.

However, this guidance is not followed consistently, and some pathologists will categorise a lesion as LCIS if they consider there are sufficient changes to make this diagnosis on a core biopsy specimen. Others will (according to the guidelines) classify both ALH and LCIS under the umbrella term LISN, even if there are sufficient features for the atypia to be regarded as LCIS. The term ALH is not used in standard core or VAB reporting, as pathologists classify these as LISN. Thus, whilst the diagnosis of LISN on core biopsy or VAB will include a mixture of cases of ALH and LCIS, if the pathologist has classified the disease as LCIS this is accepted as reliable.

In an excision specimen pathologists will (almost always be able to) distinguish ALH from LCIS, thus ALH diagnoses was derived only from excision specimens.

An LCIS diagnosis may be derived from first diagnostic procedure (if the pathologist has diagnosed it as such) or excision. As a result, LCIS diagnoses were based on a mix of procedures, both core biopsy or VAB or excision specimens, although it is anticipated that the majority were derived from the latter.

Mixed Lesions

Women with atypia can have more than one type of atypia recorded: (a) because types of atypia not infrequently co-exist and may be present in any one specimen, or (b) because different specimens during investigation may result in different diagnoses because some specific diagnoses can only be made on larger volume samples. For the latter reason, the initial diagnoses may, therefore, be revised or specified on excision. Potentially, the initial sampling may remove substantial parts of the lesion resulting in a different diagnosis on subsequent excision.

We used the following three criteria when more than one atypia type was recorded to assign an atypia type for analysis.

a) Use the most specific diagnosis if all forms of atypia present are from either the lobular or ductal group (e.g. ADH rather than AIDEP, ALH rather than LISN);

b) Use the 'worst' diagnosis if all diagnoses are from lobular or ductal group. (i.e. ADH>FEA; LCIS>ALH);

c) Use a 'mixed ductal and lobular' category for cases where both lobular and ductal atypia co-exist of any category (e.g. ADH & LCIS; LISN & AIDEP etc).

1.3 Collection and definition of cancer events

Cancer events were directly reported by the screening units by retrospective review of a list of atypia patients sent to the centres by the Sloane project up until 2013. After that annual NCRAS extracts were used to link to the Cancer registration data to identify cancer events. Centres were asked to complete a Sloane recurrence form for each event.

Cancer events were ipsilateral breast/nodal or contralateral breast/nodal DCIS or invasive events six months or more after surgical or diagnostic events due to atypia diagnosis.

For women with more than one subsequent cancer event recorded only one event was included in the analysis of cancer rates. In general, the worse diagnosis was considered (i.e. invasive rather than DCIS). In cases with a contralateral and ipsilateral diagnosis on the same date, the contralateral diagnosis was included. In cases with a contralateral and ipsilateral diagnosis on two different dates the earlier diagnosis was included. Women with a DCIS diagnosis followed by invasive cancer diagnosis were handled differently for the primary (outcome is invasive cancer only) and secondary (outcome is the first event of either DCIS or invasive cancer) analysis.

1.4 Definition of cause of death 'breast cancer'

Cause of death data were recorded in the Sloane dataset using rules by the Office for National Statistics that apply the condition or conditions entered in the lowest completed line of Part I of the Medical Certificate Cause of Death (MCCD). For this analysis, a breast cancer death was required to have a record of cause of death 'breast cancer' and a record of a breast cancer event. A cancer event classified 'distant' in conjunction with a breast cancer death was analysed as 'other cancer'.

1.5 Additional analyses

a) Investigation of cancer diagnosis by age at atypia diagnosis

We described the rates at all three time points (1 year, 3 years and 6 years) by age at atypia diagnosis to see if there are any indications of different results for women of different ages.

b) Investigation of temporal effects

The dataset contains women diagnosed with atypia from April 2003 to December 2018. Therefore, there might be some bias due to temporal effects (changes in screening technology, changes in terminology, additional atypia types, changes in diagnostic procedures, treatment and monitoring) resulting in changes in prognosis within any of the atypia types. We investigated temporal effects descriptively by looking at cancer rates for women diagnosed with atypia in different time cohorts of three 5-year periods (using date of atypia diagnosis) and comparing cancer rates and types of cancer detected at the beginning with the end of the cohort.

c) Consecutive cases only

There is some risk of selection bias if clinicians report only more interesting cases of atypia which would preclude generalisability of findings. We explored the data in 5-year intervals comparing atypia type and cancer events from consecutive cases and all cases. The definition for completeness to identify consecutive cases was based on two separate audit events:

A request was sent out to five units on 8th February 2017 to do a retrospective audit looking at patients from 2003-2006. All five units returned data for those years. Therefore, all patients with atypia from these units with a screening diagnosis from 01/04/2003 to 31/03/2006 would be classed as "Unit complete".

In 2019 a list of patients diagnosed with atypia from 01/04/2014 to 31/03/2017 was sent out to all units based on the B3 Crystal Report used by the Association of Breast Surgery (ABS)/NHSBSP Breast

Screening Audit with a covering letter asking for completion of the atypia form for each patient as well as identifying who was ineligible. All patients with atypia with a screening diagnosis 01/04/2014 to 31/03/2017 from units who have returned all of their data forms were classed as "Unit complete".

In addition, two breast units sent batches of atypia forms for all years and one unit sent atypia for the majority of years. There are emails to confirm this and patients from the units in question were also assigned the category "Unit complete".

d) Investigation of impact of management strategy

Different levels of investigation (e.g. diagnosis by core biopsy only, VAB or surgical procedure) may have an impact on cancer prognosis. We explored cancer rates following diagnosis of atypia by three levels of management.

The categories of different management levels reflect the size of the sample taken for diagnostic purposes based on the following rationale:

The diagnostic pathway for atypia following a recall from screening typically includes an initial diagnostic procedure (standard core or vacuum assisted biopsy (VAB)), followed by a second diagnostic procedure either surgical excision or a second vacuum assisted specimen. The initial procedure is diagnostic, while the second procedure includes a greater proportion of the lesion for diagnostic purposes and may even excise the whole area. This second VAB is, therefore, referred to as vacuum assisted excision (VAE). A VAE typically includes a larger sample than VAB and a surgical procedure often samples the largest volume. Women with an atypia diagnosis will have had different numbers and types of procedures undertaken along their diagnostic pathway based on the year of their diagnosis (i.e. before or after UK guidelines were published for management of B3 lesions (Pinder et al. 2018)), preference, availability of methods and whether they could technically be sampled by VAE.

The procedures vary in their diagnostic accuracy (because of the difference in amount of tissue received by the pathologist) as well as, potentially, their prognostic ability (as the recurrence rate may be affected by the amount of tissue sampled, and thus the extent of the area of atypia removed, during the investigation process). The three management strategies include women with:

1. Only one initial diagnostic procedure (standard core or VAB);
2. An initial diagnostic procedure (standard core or VAB) and a second vacuum assisted procedure (recorded as a (therapeutic) VAB or VAE) (no surgical procedure);
3. An initial diagnostic procedure (standard core or VAB) and a minimum of one surgical procedure (+/- additional VAB/VAE).

Reference:

Pinder SE, Shaaban A, Deb R, Desai A, Gandhi A, Lee AHS, Pain S, Wilkinson L, Sharma N. NHS Breast Screening multidisciplinary working group guidelines for the diagnosis and management of breast lesions of uncertain malignant potential on core biopsy (B3 lesions). Clin Radiol. 2018;73(8):682-92. doi: 10.1016/j.crad.2018.04.004.

e) Investigation of cancer diagnoses within 1 year of atypia

Cancers typically take longer than 12 months to develop. Cancer diagnoses within 12 months after the atypia diagnosis likely represent missed cancers due to under sampling. We performed an analysis to explore the impact of excluding cancers diagnosed within one year of a diagnosis from the main analysis.

1.6 Options explored how to add age at diagnosis into the model

Four methods of including age at diagnosis were explored: grouped (46 to 55, 56 to 60, 61 to 65, 66 to 70, 71 to 95), continuous linear, continuous linear and quadratic, and a cubic spline. Including age as a continuous, linear term was the best method, having better model fit statistics than the equivalent models for group, linear and quadratic and spline, showing the extra complexity to be unnecessary.

1.7 Flexible parametric model choice – rationale

We analysed time-to-event data with competing risks, considering the first event that happened to each person only; In the main model the competing causes were a diagnosis of invasive breast cancer or death. We also modelled with invasive cancer split into ipsilateral and contralateral, and with invasive cancer and DCIS combined. There are two methods that are used: cause-specific hazards and subdistribution hazards. Putter et al. (2020) gives a succinct summary of the two methods. The cause-specific hazards method models the hazard over time for each cause separately, estimating the hazard of someone who has not yet had an event having an event of that cause. The subdistribution hazards method (Fine and Gray, 1999) evaluates the hazard of an event of the cause of interest amongst those who have yet to have an event of the cause of interest; which it does by including those who have had an event of another cause in with those who have yet to have an event in the “risk set”, even though they are unable to have an event of the cause of interest at that time.

The purpose of the analysis may affect the choice of method. Lau et al. (2009) suggest that when looking at the “etiology” of the different causes then cause-specific hazards are better, whereas if trying to predict someone’s risk then subdistribution hazards are preferred. We chose to use the cause-specific hazards method, because we wanted to explore how each cause affects the time to event specifically. However, we also decided to run the chosen model in the subdistribution framework, since prediction of a person’s risk of an event is an important result for the study. We present the cumulative incidence function from the main model (evaluated at 1 year, 3 years and 6 years after atypia diagnosis) fitted with both cause-specific hazards and subdistribution hazards.

We chose to use the method of Hinchliffe and Lambert (2013b) for our main analysis, which enables us to model a baseline hazard function for each cause separately, each with their own shape. This involves using a dataset where each case is included once for each cause (twice for models with death and invasive cancer and three times when cancer is split into ipsilateral and contralateral). The method was run in Stata using the stpm2 package (Lambert and Royston, 2009). Variables indicating which cause the row relates to were added as main effects and time varying effects with 3 degrees of freedom so that the flexible parametric model can model the baseline hazard for each cause with a restricted cubic spline. The potential explanatory effects were added as main effects on the log cumulative hazard scale, by using scale(hazard) in the stpm2 command. Using the log cumulative hazard scale implies that the variables are being added into the model using proportional hazards.

We plotted Kaplan-Meier survival curves for the event death or invasive cancer (combining the two causes into one) stratified by the groups of each explanatory variable in the chosen model, and found nothing to suggest that assuming proportional hazards was unreasonable. The cause-specific cumulative incidence functions for various values of the explanatory variables in the model were calculated from the model using the postestimation command stpm2cif (Hinchliffe and Lambert, 2013a). The command produces estimates and confidence intervals for the cause-specific cumulative incidence function and the cause-specific hazard function.

A subdistribution hazards model was run using the same covariates as that of the chosen cause-specific hazards model. The model was run in R using the Survival package; using the finegray function to adapt the dataset so that a Fine-Gray model can be run using the coxph function. This used invasive cancer as the event of interest and death as the competing risk, remaining in the "risk set". The cumulative incidence function for invasive cancer was estimated using the survfit function, using the "log-log" type of confidence intervals.

Interpretation of model coefficients in competing risks models.

Austin and Fine (2017) remind that in a competing risks model using cause-specific hazards the model coefficients only tell us about the effect of the explanatory variable on the cause-specific hazard function, and not about its effect on the cumulative incidence function. This is because the cumulative incidence function for each cause is dependent on the cause-specific hazard functions for all causes, not just its own cause. Therefore, care must be taken in interpretating the cause-specific hazards model coefficients. The cause-specific hazard ratios we give show the effect of the explanatory variable on that cause's cause-specific hazard function only.

We have presented the cause-specific cumulative incidence functions for each cause, evaluated at given time points for combinations of values for the three explanatory variables in the model. The effect of each explanatory variable on the cumulative incidence functions can be seen by considering the evaluations in these tables.

Austin and Fine also state that for the subdistribution hazard model the hazard ratio does not show the size of the effect of the explanatory variable on the cumulative incidence function, like it does on the subdistribution hazard function.

Therefore, we advise care when evaluating model coefficients and hazard ratios from competing risks models. For cause-specific hazards models in particular there is no single number that can be used to evaluate the effect of an explanatory variable on the cumulative incidence function.

References

Peter C. Austin and Jason P. Fine (2017) Practical recommendations for reporting Fine-Gray model analyses for competing risk data. Statistics in Medicine.36:4391–4400. DOI: 10.1002/sim.7501

Lau B, Cole SR, Gange SJ. Competing risk regression models for epidemiologic data. Am J Epidemiol. 2009 Jul 15;170(2):244-56. doi: 10.1093/aje/kwp107. Epub 2009 Jun 3. PMID: 19494242; PMCID: PMC2732996.

Hein Putter, Martin Schumacher, Hans C. van Houwelingen (2020) On the relation between the cause-specific hazard and the subdistribution rate for competing risks data: The Fine–Gray model revisited. Biometrical Journal. 62:790–807. DOI: 10.1002/bimj.201800274

Jason P Fine and Robert J Gray (1999) “A proportional hazards model for the subdistribution of a competing risk.” Journal of the American Statistical Association, 94:496-509.

Sally R Hinchliffe and Paul C Lambert (2013a) “Extending the flexible parametric survival model for competing risks” The Stata Journal 13, Number 2, pp. 344–355

Sally R Hinchliffe and Paul C Lambert (2013b) “Flexible parametric modelling of cause-specific hazards to estimate cumulative incidence functions” BMC Medical Research Methodology 2013, 13:13, http://www.biomedcentral.com/1471-2288/13/13

Paul C. Lambert and Patrick Royston (2009) “Further development of flexible parametric models for survival analysis” The Stata Journal, 9:2, p 265-290.

2. RESULTS

2.1 Study population

Figure S1 Flow diagram of study population

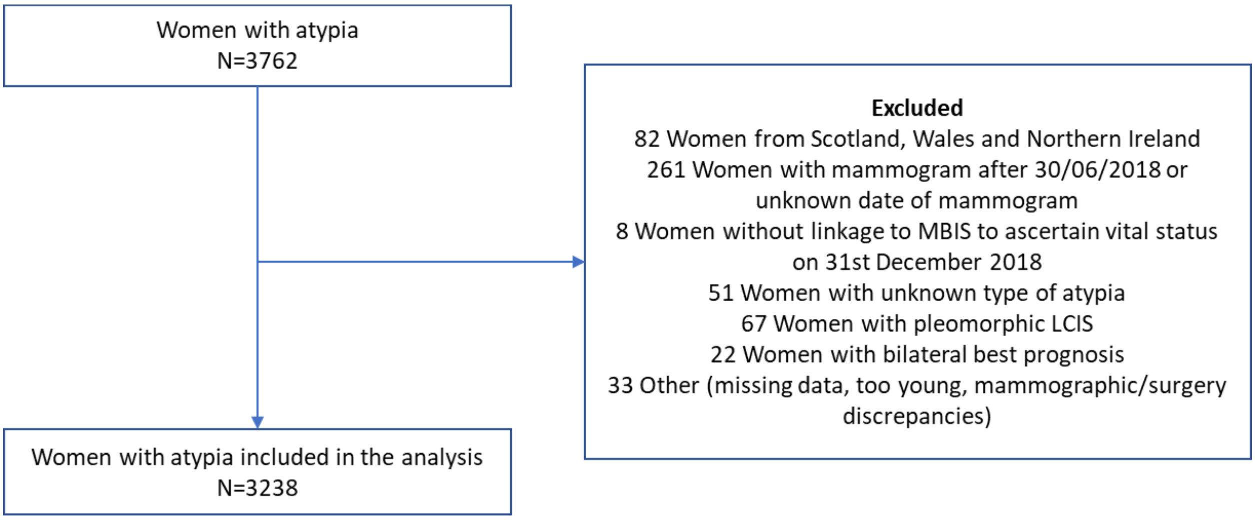

2.2 Characteristics of women and their atypia in the Sloane atypia cohort

Table S1 Descriptive statistics of all women with atypia for the study period April 2003 to June 2018

	**All atypia**	**ADH (including AIDEP)**	**FEA**	**LISN (LN)**				**Mixed ductal and lobular**
				ALH on excision	LCIS (on initial diagnostic procedure or on excision)	LISN unspecified (only LN/LISN reported or ALH on core biopsy)	All LISN	
n (%)	3238	1350* (41.7)	403 (12.4)	77 (2.4)	482 (14.9)	542 (16.7)	1101 (34.0)	384 (11.9)
Age at atypia diagnosis in years								
mean (SD)	55.63 (7.28)	56.55 (7.62)	54.5 (6.98)	55.06 (6.51)	55.03 (7.12)	55.51 (7.17)	55.27 (7.10)	54.58 (6.48)
range	46; 95	46; 95	46; 78	47; 74	46; 83	46; 78	46; 83	46; 81
Number of screening round at which atypia was diagnosed								
median	2.0	2.0	1.0	1.0	1.0	2.0	1.0	1.0
IQR	1.0; 3.0	1.0; 4.0	1.0; 3.0	1.0; 2.5	1.0; 3.0	1.0; 3.0	1.0; 3.0	1.0; 3.0
Range	1.0; 17.0	1.0; 17.0	1.0; 12.0	1.0; 8.0	1.0; 11.0	1.0; 11.0	1.0; 11.0	1.0; 10.0
missing	490	175	101	14	58	78	150	64
Time of follow-up in years								
mean (SD)	5.90 (3.96)	6.0 (4.10)	4.21 (2.50)	5.36 (4.22)	6.37 (3.65)	6.59 (4.32)	6.41 (4.04)	5.83 (4.01)
median	4.42	4.40	3.83	3.91	5.91	4.78	5.32	4.30
IQR	2.86; 8.35	2.86; 8.77	2.52; 5.19	2.40; 6.92	3.50; 8.46	2.86; 10.47	3.07; 9.43	2.73; 7.94
range	0.51; 15.72	0.53; 15.72	0.59; 15.63	0.66; 15.71	0.51; 15.63	0.52; 15.72	0.51; 15.72	0.54; 15.72
Level of management n (%)								
• One diagnostic procedure only (standard core or VAB)	477 (14.7)	143 (10.6)	75 (18.6)	2 (2.6)	100 (20.7)	134 (24.7)	236 (21.4)	23 (6.0)
• Diagnostic procedure plus therapeutic VAB/VAE	964 (29.8)	414 (30.7)	152 (37.7)	24 (31.2)	110 (22.8)	148 (27.3)	282 (25.6)	116 (30.2)
	1797 (55.5)	793 (58.7)	176 (43.7)	51 (66.2)	272 (56.4)	260 (48.0)	583 (53.0)	245 (63.8)

• Diagnostic procedure plus surgical procedure (+/- therapeutic VAB/VAE)								
Management strategy n (%) (not mutual exclusive)								
Diagnostic open biopsy	1488 (46.0)	667 (49.4)	158 (39.2)	44 (57.1)	207 (42.9)	207 (38.2)	458 (41.6)	205 (53.4)
Excision	514 (15.9)	223 (16.5)	25 (6.2)	8 (10.4)	86 (17.8)	103 (19.0)	197 (17.9)	69 (18.0)
Mastectomy	15 (0.5)	0	1 (0.2)	0	5 (1.0)	7 (1.3)	12 (1.1)	2 (0.5)
Multiple operations	55 (1.7)	15 (1.1)	4 (1.0)	0	12 (2.5)	18 (3.3)	30 (2.7)	5 (1.3)
Other surgery	0	0	0	0	0	0	0	0
Axillary surgery	24 (0.7)	5 (0.4)	0	2 (2.6)	3 (0.6)	11 (2.0)	16 (1.5)	3 (0.8)
Endocrine treatment	19 (0.6)	1 (0.1)	0	1 (1.3)	6 (1.2)	9 (1.7)	16 (1.5)	2 (0.5)
Radiotherapy	6 (0.2)	3 (0.2)	0	0	2 (0.4)	1 (0.2)	3 (0.3)	0
Radiotherapy (unrecorded)	3 (0.1)	0	1 (0.2)	0	2 (0.4)	0	2 (0.2)	0
Other therapy	6 (0.2)	6 (0.4)	0	0	0	0	0	0
No surgery	1444 (44.6)	559 (41.4)	227 (56.3)	26 (33.8)	210 (43.6)	282 (52.0)	518 (47.0)	140 (36.5)
No further adjuvant therapy	3103 (95.8)	1237 (91.6)	402 (99.8)	76 (98.7)	474 (98.3)	532 (98.2)	1082 (98.3)	382 (99.5)
Surgical procedure not specified	0	0	0	0	0	0	0	0

*This includes 326 (10.1%) women who received an AIDEP diagnosis without an ADH diagnosis

Table S2 Number of women with atypia and characteristics of subsequent cancers detected following atypia diagnosis by atypia type for three time periods separately

	**All atypia**	**ADH**	**FEA**	**LISN (LN)**				**Mixed ductal and lobular**
				**ALH on excision**	**LCIS**	**LISN unspec**	**All LISN**	
**2003 to 2007**								
Number of women with atypia n (%)	534	244 (45.7)	14 (2.6)	12 (2.2)	67 (12.5)	127 (23.8)	206 (38.6)	70 (13.1)

Number of women with a cancer detected n (%)	88 (16.5)	30 (12.3)	3 (21.4)	3 (25.0)	11 (16.4)	24 (18.9)	38 (18.4)	17 (24.3)
Invasive cancer n (%)	75 (85.2)	26 (86.7)	3 (100)	3 (100)	9 (81.8)	19 (79.2)	31 (81.6)	15 (88.2)
Non-invasive cancer (DCIS) n (%)	9 (10.2)	4 (13.3)	0	0	1 (9.1)	3 (12.5)	4 (10.5)	1 (5.9)
**2008 to 2012**								
Number of women with atypia n (%)	690	276 (40.0)	50 (7.2)	10 (1.4)	172 (24.9)	109 (15.8)	291 (42.2)	73 (10.6)
Number of women with a cancer detected n (%)	53 (7.7)	23 (8.3)	1 (2.0)	2 (20.0)	10 (5.8)	9 (8.3)	21 (7.2)	8 (11.0)
Invasive cancer n (%)	47 (88.7)	19 (82.6)	1 (100)	2 (100)	9 (90.0)	9 (100)	20 (95.2)	7 (87.5)
Non-invasive cancer (DCIS) n (%)	6 (11.3)	4 (17.4)	0	0	1 (10.0)	0	1 (4.8)	1 (12.5)
**2013 to 2018**								
Number of women with atypia n (%)	2014	830 (41.2)	339 (16.8)	55 (2.7)	243 (12.1)	306 (15.2)	604 (30.0)	241 (12.0)
Number of women with a cancer detected n (%)	32 (1.6)	12 (1.4)	9 (2.7)	0	2 (0.8)	3 (1.0)	5 (0.8)	6 (2.5)
Invasive cancer n (%)	19 (59.4)	9 (75.0)	4 (44.4)	0	2 (100)	1 (33.3)	3 (60.0)	3 (50.0)
Non-invasive cancer (DCIS) n (%)	12 (37.5)	3 (25.0)	5 (55.6)	0	0	1 (33.3)	1 (20.0)	3 (50.0)

Figure S2 Number of atypia with microcalcifications present or absent by year

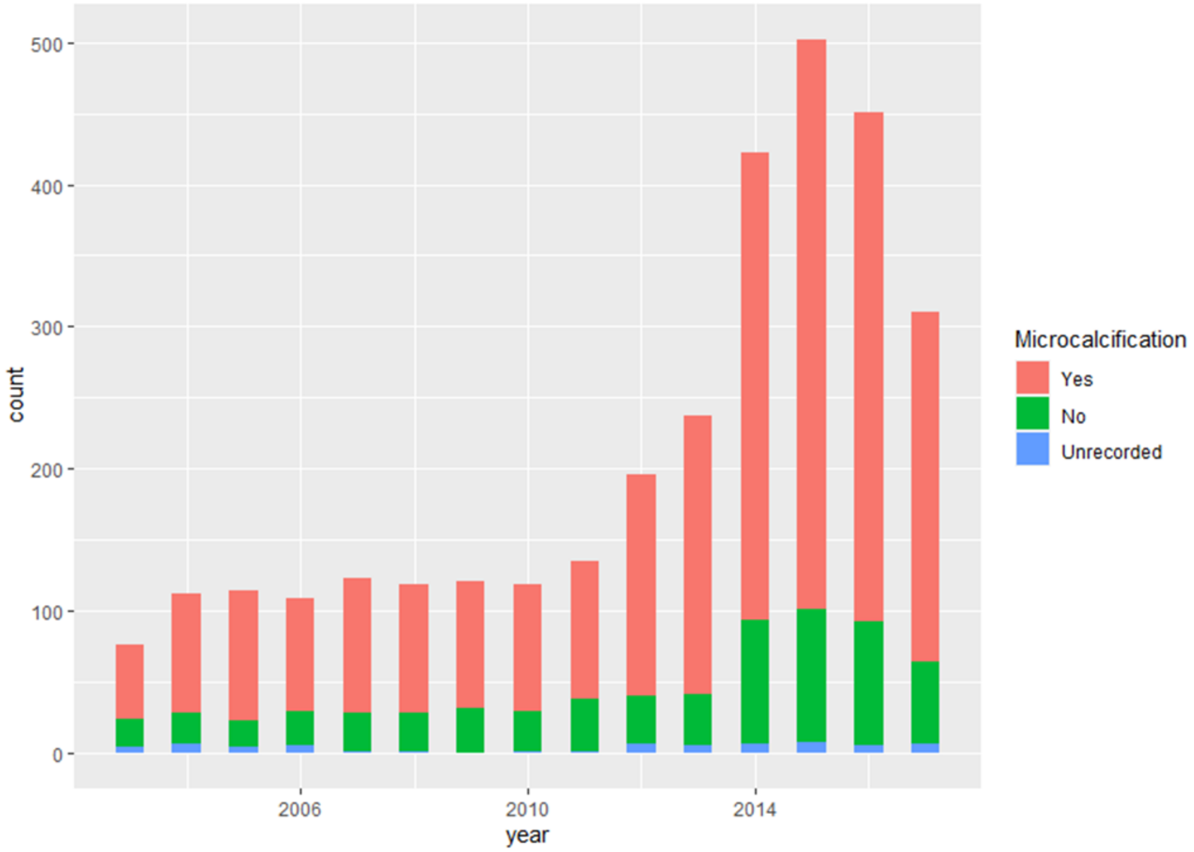

2.3 Subsequent events following atypia

Table S3 Characteristics of subsequent invasive and non-invasive cancers by atypia type and number of deaths

	All atypia	ADH/AIDEP	FEA	LISN (LN)				Mixed ductal and lobular
				ALH on excision	LCIS	Unspecified LISN	All LISN	
Number of women n	3238	1350† (41.7)	403 (12.4)	77 (2.4)	482 (14.9)	542 (16.7)	1101 (34.0)	384 (11.9)
Number of women with breast cancer n (%)	168 (5.2)*	65 (4.8)	13 (3.2)	5 (6.5)	22 (4.6) (*)	33 (6.1) (*)	60 (5.4)*	30 (7.8)
Deaths from breast cancer n (%)	10‡ (5.8)	3 (4.6)	0	0	3 (13.6)	3† (9.1)	6‡ (10.0)	1 (3.3)
Deaths from other causes in breast cancer women								
Other cancer n (%)	1 (0.6)	0	0	0	0	0	0	1 (3.3)
Non-cancer n (%)	3 (1.7)	1 (1.5)	0	1 (20.0)	0	1 (3.0)	2 (3.3)	0
Cancer (unknown type) n (%)	1 (0.6)	1 (1.5)	0	0	0	0	0	0
Deaths from other causes in atypia women								
Other cancer n (%)	35 (20.2)	18 (27.7)	1 (7.7)	1 (20.0)	2 (9.1)	8 (24.2)	11 (18.3)	5 (16.7)
Non-cancer n (%)	51 (29.5)	24 (36.9)	4 (30.8)	2 (40.0)	7 (31.8)	9 (27.3)	18 (30.0)	5 (16.7)
Cancer (unknown type) n (%)	2 (1.2)	1 (1.5)	0	0	1 (4.5)	0	1 (1.7)	0
Invasive cancer n (%)	141** (83.9)	54 (83.1)	8 (61.5)	5 (100)	20 (90.9)	29 (87.9)	54 (90.0)	25** (83.3)
Site								
Ipsilateral cancer n (%)	82 (58.2)	29 (53.7)	4 (50.0)	3 (60.0)	12 (60.0)	17 (58.6)	32 (59.3)	17 (68.0)
Contralateral cancer n (%)	59 (41.8)	25 (46.3)	4 (50.0)	2 (40.0)	8 (40.0)	12 (41.4)	22 (40.7)	8 (32.0)
Grade n (%)								
1	25 (17.7)	9 (16.7)	1 (12.5)	2 (40.0)	3 (15.0)	3 (10.3)	8 (14.8)	7 (28.0)
2	69 (48.9)	27 (50.0)	5 (62.5)	2 (40.0)	12 (60.0)	14 (48.3)	28 (51.9)	9 (36.0)
3	28 (19.9)	15 (27.8)	1 (12.5)	1 (20.0)	2 (10.0)	4 (13.8)	7 (13.0)	5 (20.0)
Unrecorded	19 (13.5)	3 (5.6)	1 (12.5)	0	3 (15.0)	8 (27.6)	11 (20.4)	4 (16.0)
Size in mm								
Mean (SD)	21.17 (18.33)	19.76 (16.40)	30.67 (43.83)	11.67 (3.76)	27.39 (20.57)	20.85 (13.74)	23.05 (17.05)	17.84 (13.38)
Median	15.0	15.0	14.0	10.0	19.0	19.5	18.0	12.0
IQR	9.75; 27.25	10.0; 24.75	13.25; 14.75	9.5; 13.0	11.25; 46.0	10.25; 28.0	10.0; 30.0	8.5; 22.0
Range	3.0; 120.0	3.0; 100.0	8.0; 120.0	9.0; 16.0	5.0; 70.0	4.0; 59.0	4.0; 70.0	4.0; 46.0
≤20mm n (%)	77 (54.6)	35 (64.8)	5 (62.5)	3 (60.0)	10 (50.0)	10 (34.5)	23 (42.6)	14 (56.0)
>20mm to ≤50mm n (%)	32 (22.7)	14 (25.9)	0	0	4 (20.0)	9 (31.0)	13 (24.1)	5 (20.0)

>50mm n (%)	7 (5.0)	1 (1.9)	1 (12.5)	0	4 (20.0)	1 (3.4)	5 (9.3)	0
Unrecorded n (%)	25 (17.7)	4 (7.4)	2 (25.0)	2 (40.0)	2 (10.0)	9 (31.0)	13 (24.1)	6 (24.0)
Nodal status n (%)								
0 nodes	84 (59.6)	33 (61.1)	5 (62.5)	3 (60.0)	13 (65.0)	14 (48.3)	30 (55.6)	16 (64.0)
1,2 or 3 nodes	22 (15.6)	12 (22.2)	0	0	2 (10.0)	4 (13.8)	6 (11.1)	4 (16.0)
>3 nodes	7 (5.0)	3 (5.6)	2 (25.0)	0	2 (10.0)	0	2 (3.7)	0
Unrecorded	28 (19.9)	6 (11.1)	1 (12.5)	2 (40.0)	3 (15.0)	11 (37.9)	16 (29.6)	5 (20.0)
Hormone receptor status n (%)								
Estrogen positive	108 (76.6)	39 (72.2)	7 (87.5)	4 (80.0)	19 (95.0)	21 (72.4)	44 (81.5)	18 (72.0)
Estrogen negative	10 (7.1)	8 (14.8)	0	0	0	1 (3.4)	1 (1.9)	1 (4.0)
Estrogen not known / unrecorded	23 (16.3)	7 (13.0)	1 (12.5)	1 (20.0)	1 (5.0)	7 (24.1)	9 (16.7)	6 (24.0)
Progesteron positive	47 (33.3)	14 (25.9)	2 (25.0)	3 (60.0)	10 (50.0)	8 (27.6)	21 (38.9)	10 (40.0)
Progesteron negative	10 (7.1)	5 (9.3)	0	1 (20.0)	0	4 (13.8)	5 (9.3)	0
Progesteron not known / unrecorded	84 (59.6)	35 (64.8)	6 (75.0)	1 (20.0)	10 (50.0)	17 (58.6)	28 (51.9)	15 (60.0)
HER-2 positive	15 (10.6)	5 (9.3)	0	0	3 (15.0)	2 (6.9)	5 (9.3)	5 (20.0)
HER-2 negative	89 (63.1)	35 (64.8)	5 (62.5)	4 (80.0)	16 (80.0)	16 (55.2)	36 (66.7)	13 (52.0)
HER-2 not known / unrecorded	37 (26.2)	14 (25.9)	3 (37.5)	1 (20.0)	1 (5.0)	11 (37.9)	13 (24.1)	7 (28.0)
Non-invasive cancer (DCIS) n (%)	27 (16.1)	11 (16.9)	5 (38.5)	0	2 (9.1)	4 (12.1)	6 (10.0)	5 (16.7)
Site								
Ipsilateral n (%)	20 (47.1)	9 (81.8)	3 (60.0)	0	2 (100.0)	2 (50.0)	4 (66.7)	4 (80.0)
Contralateral n (%)	7 (25.9)	2 (18.2)	2 (40.0)	0	0	2 (50.0)	2 (33.3)	1 (20.0)
Grade n (%)								
1	4 (14.8)	2 (18.2)	1 (20.0)	0	0	1 (25.0)	1 (16.7)	0
2	6 (22.2)	4 (36.4)	1 (20.0)	0	0	1 (25.0)	1 (16.7)	0
3	12 (44.4)	4 (36.4)	3 (60.0)	0	1 (50.0)	1 (25.0)	2 (33.3)	3 (60.0)
Unrecorded	5 (18.5)	1 (9.1)	0	0	1 (50.0)	1 (25.0)	2 (33.3)	2 (40.0)
Size in mm								
Mean (SD)	16.62 (17.02)	13.75 (9.74)	NA	NA	NA	27.0 (37.32)	27.75 (30.51)	9.67 (9.61)
Median	10.0	10.0	NA	NA	NA	8.0	19.0	8.0
IQR	7.0; 20.0	7.0; 15.5	NA	NA	NA	5.5.; 39.0	6.75; 40.0	4.5; 14.0
Range	1.0; 70.0	7.0; 35.0	NA	NA	NA	3.0; 70.0	3.0; 70.0	1.0; 20.0
≤20mm n (%)	13 (48.1)	7 (63.6)	1 (20.0)	0	0	2 (50.0)	2 (33.3)	3 (60.0)
>20mm to ≤50mm n (%)	2 (7.4)	1 (9.1)	0	0	1 (50.0)	0	1 (16.7)	0
>50mm n (%)	1 (3.7)	0	0	0	0 (	1 (25.0)	1 (16.7)	0
Unrecorded n (%)n (%)	11 (40.7)	3 (27.3)	4 (80.0)	0	1 (50.0)	1 (25.0)	2 (33.3)	2 (40.0)

*An additional 2 women had a recorded distant cancer, but no breast cancer recorded.
** This includes one woman with an invasive cancer recorded but no date of detection, who is therefore not included in the analysis of cancer rates at 1, 3, and 6 years following atypia diagnosis.
†This includes 326 (10.1%) women who received an AIDEP diagnosis without an ADH diagnosis
‡No death certificate only breast cancer death occurred, however, one additional woman with a distant cancer but no record of a breast cancer had breast cancer as cause of death

Table S4 Distribution of grade for subsequent invasive cancers compared to published figures

	All invasive cancers (n=141)	Cancers within 3 years (n=40)	Screen detected (Allgood 2011*) (n=7737)	Symptomatic detected (Allgood 2011*) (n=11674)
Grade 1	25 (17.7%)	5 (12.5%)	2045 (26.4%)	1099 (9.4%)
Grade 2	69 (48.9%)	18 (45.0%)	3038 (39.3%)	2719 (23.3%)
Grade 3	28 (19.9%)	9 (22.5%)	1327 (17.2%)	3898 (33.4%)
Unrecorded	19 (13.5%)	8 (20.0%)	1327 (17.2%)	2958 (25.3%)

*Allgood PC, Duffy SW, Kearins O, O'Sullivan E, Tappenden N, Wallis MG, Lawrence G. Explaining the difference in prognosis between screen-detected and symptomatic breast cancers. Br J Cancer. 2011 May 24;104(11):1680-5. doi: 10.1038/bjc.2011.144.

Table S5 Location of 22 ipsilateral invasive cancers 3 years post atypia diagnosis by atypia type

		**ADH**	**FEA**	**LISN**	**Mixed**
Location of subsequent invasive cancer	At or adjacent to site of atypia	3	0	3	2
	Some distance from atypia	1	0	1	0
	Other	0	0	0	0
	Unrecorded	4	2	3	3

2.4 Missed cancers at atypia diagnosis

Figure S3 Proportion of diagnostic management options performed by year

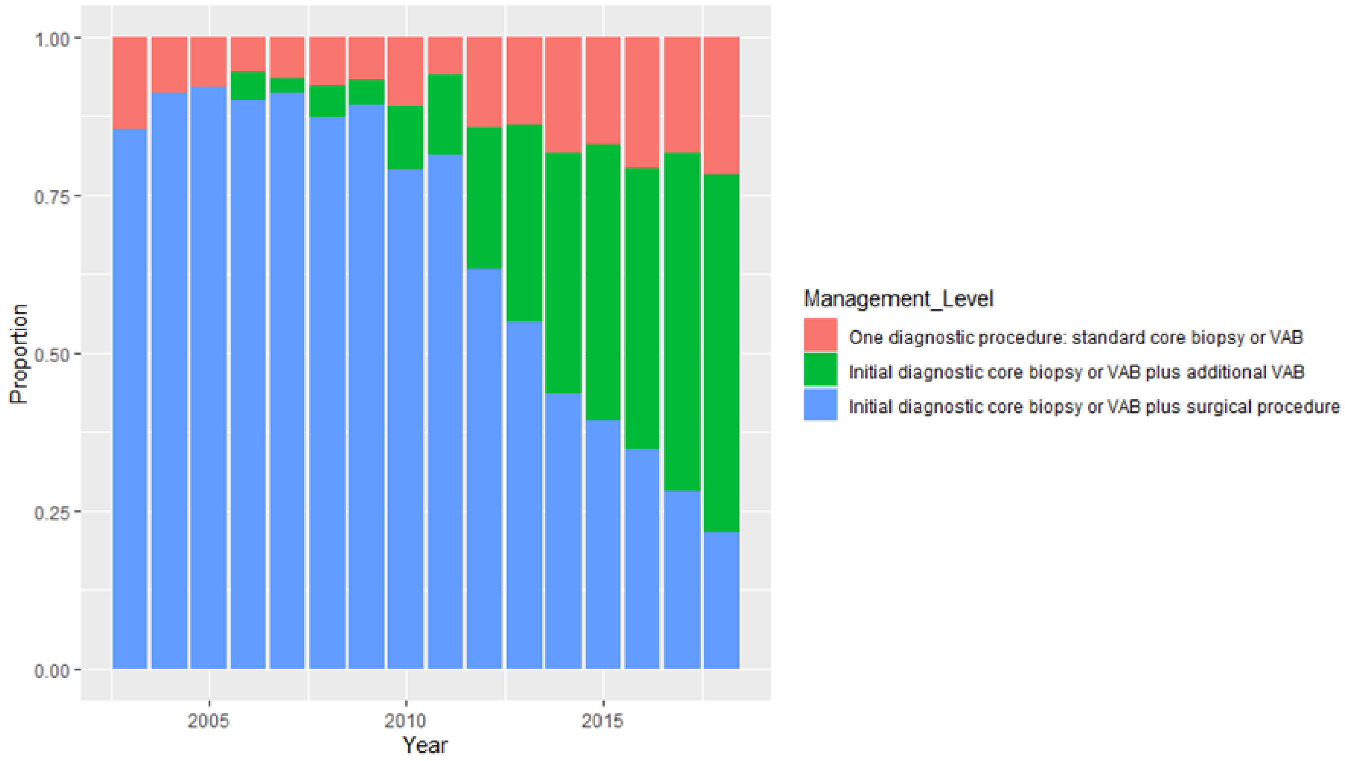

Table S6 Invasive cancers per 1000 women with atypia at 1 year and 3 years post atypia diagnosis estimated from CIF by management strategy and site of invasive cancers separately for atypia types

	**Cancers per 1000 women at 1 year**		**Cancer per 1000 women at 3 years**	
	**Ipsilateral**	**Contralateral**	**Ipsilateral**	**Contralateral**
**Following a diagnosis of ADH**				
'Diagnostic' needle biopsy only with no second procedure	0	0	0	0
Second line vacuum biopsy/excision and no surgery	0	2.54 (0.25,13.5)	5.95 (1.21,20.1)	5.83 (1.18,19.8)
Management involves diagnostic surgical excision	0	0	8.21 (3.45,17.1)	8.08 (3.4,16.9)
**Following a diagnosis of FEA**				
'Diagnostic' needle biopsy only with no second procedure	0	0	0	0
Second line vacuum biopsy/excision and no surgery	0	0	7.87 (0.711,39.3)	10.10 (0.893,49.7)
Management involves diagnostic surgical excision	0	0	6.54 (0.601,33)	0
**Following a diagnosis of LISN**				
'Diagnostic' needle biopsy only with no second procedure	0	0	9.89 (1.96,32.8)	0
Second line vacuum biopsy/excision and no surgery	0	0	0	3.91 (0.374,20.3)
Management involves diagnostic surgical excision	0	0	8.83 (3.38,19.6)	11.10 (4.64,23)
**Following a diagnosis of mixed atypia**				
'Diagnostic' needle biopsy only with no second procedure	0	0	0	0
Second line vacuum biopsy/excision and no surgery	0	0	0	0
Management involves diagnostic surgical excision	8.26 (1.67,27.4)	0	21.80 (8.23,47.4)	8.56 (1.73,28.4)

2.5 Cancers at 3- and 6-years post atypia and long-term risk

Table S8 Invasive cancer rates per 1000 women with atypia at 1 year, 3 years and 6 years post atypia diagnosis by age group estimated from CIF

**Age at atypia diagnosis**	**1 year**	**3 years**	**6 years**
≤55 years	1.03 (0.222,3.6)	9.82 (5.95,15.4)	39.8 (29.3,52.7)
56 to 60 years	0	20.4 (9.61,38.3)	60.3 (36.2,92.9)
61 to 65 years	2.91 (0.284,15.3)	26.2 (12.3,49)	66.3 (38.7,104)
66 to 70 years	0	6.28 (1.28,21)	24.4 (9.94,50.1)
>70 years	0	52 (19.2,109)	64.9 (26.3,128)

Figure S4. Cumulative incidence function for invasive cancer and death from the main model, evaluated at 1 year, 3 years, and 6 years since diagnosis with atypia; by age at diagnosis, year of diagnosis and women's breast background parenchymal density (high figure 3a, low figure 3b).

a
High
density

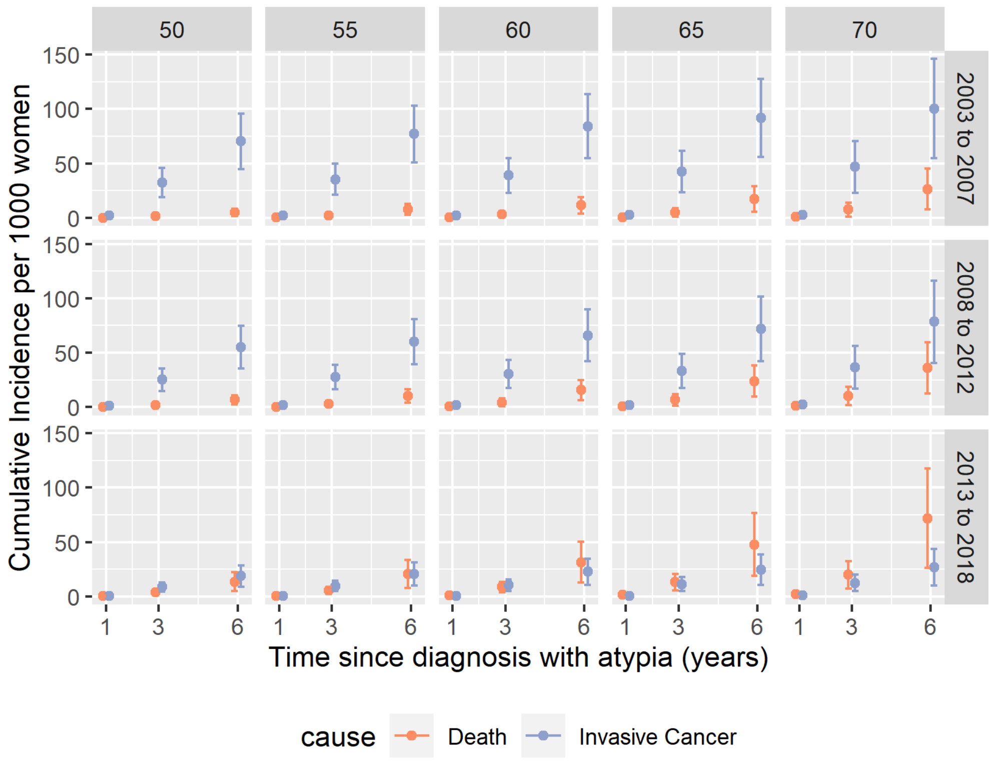

b
Low
density

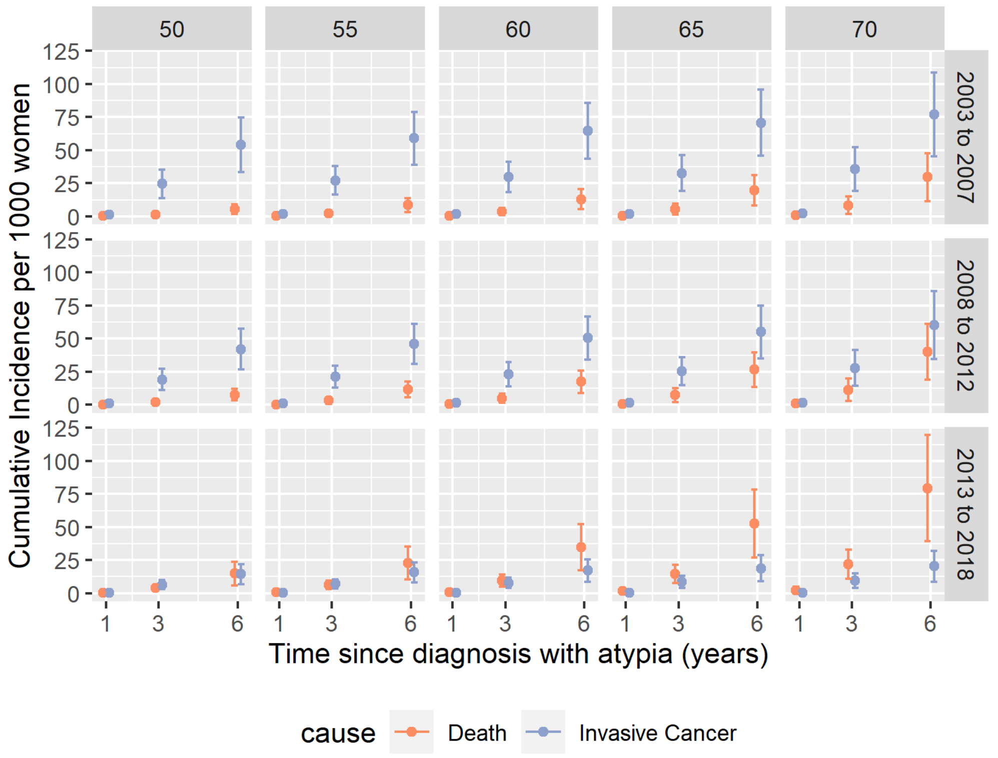

Table S9 Invasive cancer rates at 3 years post atypia by atypia type estimated from CIF

	ADH	FEA	LISN	Mixed
Invasive cancer per 1000 women with atypia (95% CI)	13.6 (8.1,21.6)	9.47 (2.62,25.9)	14.3 (8.23,23.5)	20.8 (9.21,40.6)

Table S10 Invasive cancer rates at 1 year, 3 years and 6 years post atypia diagnosis per 1000 women estimated from CIF for 3 time periods under different scenarios

Calendar year at atypia diagnosis	N atypia cases	1 year		3 years		6 years	
		Cancer cases	Cancers per 1000 women (95% CI)	Cancer cases	Cancers per 1000 women (95% CI)	Cancer cases	Cancers per 1000 women (95% CI)
2003-2018	3238	3	0.95 (0.28,2.69)	40	14.2 (10.3,19.1)	94	45.0 (36.3,55.1)
2003-2007	534	0	0	13	24.3 (13.7, 40.1)	36	67.4 (48.2, 90.8)
2008-2012	690	2	2.9 (0.61, 9.94)	17	24.6 (14.9, 38.3)	40	58.0 (42.2, 77.1)
2013-2018	2014	1	0.51 (0.055, 2.89)	10	6.0 (3.09, 10.9)	18	-
2013-2015 (at least 3 years follow-up)	1161	1	0.861 (0.09, 4.8)	7	6.03 (2.7, 12.0)	-	-
2013-2014 (at least 4 years follow-up)	659	1	1.52 (0.15, 8.2)	3	4.55 (1.3, 12.6)	-	-

Table S11 Invasive cancer rates at 1 year, 3 years and 6 years post atypia diagnosis per 1000 women estimated from CIF for 3 time periods excluding women with FEA

Calendar year at atypia diagnosis	N atypia cases	1 year		3 years		6 years	
		Cancer cases	Cancers per 1000 women (95% CI)	Cancer cases	Cancers per 1000 women (95% CI)	Cancer cases	Cancers per 1000 women (95% CI)
2003-2007	520	0	0	13	25.0 (14.0, 41.1)	33	63.5 (44.7, 86.6)
2008-2012	640	2	3.1 (0.65, 10.7)	17	26.6 (16.1, 41.2)	39	60.9 (44.2, 81.3)
2013-2018	1675	1	0.62 (0.066, 3.47)	7	4.75 (2.1, 9.50)	-	-

Table S12 Invasive cancer rates at 1 year, 3 years and 6 years post atypia diagnosis per 1000 women estimated from CIF for 3 time periods for consecutive cases only

Calendar year at atypia diagnosis	N atypia cases	1 year		3 years		6 years	
		Cancer cases	Cancers per 1000 women (95% CI)	Cancer cases	Cancers per 1000 women (95% CI)	Cancer cases	Cancers per 1000 women (95% CI)
2003-2007	172	0	0	5	29.1 (10.9, 62.6)	15	87.2 (51.1, 135.2
2008-2012	215	2	9.3 (1.87, 30.7)	6	27.9 (11.5, 56.6)	12	55.8 (30.5, 92.1)
2013-2018	1281	1	0.79 (0.083, 4.36)	3	2.5 (0.72, 7.03)	-	-

2.6 Mode of detection of subsequent cancers

Figure S5 Number of cancers (invasive and DCIS) over time since atypia diagnosis by mode of detection

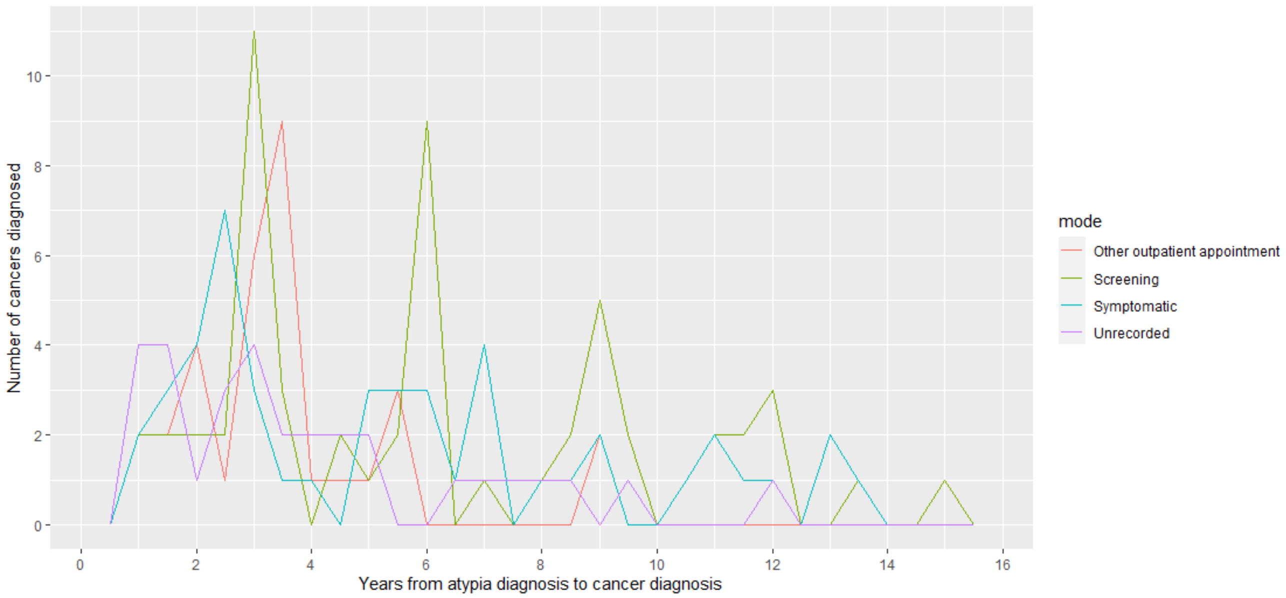

3. Modelling

3.1 Modelling of cancer rates using the cause specific hazard method

Main analysis

Table S13 Model selection for models with invasive cancer and death as causes of outcome.

Number of parameters (p), sample size (n), model log likelihood (Loglik), Akaike's Information criterion (AIC), Bayesian Information Criterion (BIC). Age at atypia diagnosis included grouped (group), continuous linear (cts), continuous linear and quadratic, and as a cubic polynomial spline (spline). Adding continuous linear age as a time varying covariate (tvc) was also explored. The sample size is 6476 because each person contributes two rows to the dataset, one for each cause.

Model	p	n	Loglik	AIC	BIC
No covariates	8	6476	-970.42	1956.84	2011.05
Age (group)	16	6476	-944.64	1921.28	2029.69
Age (cts)	10	6476	-948.79	1917.57	1985.33
Age (cts, tvc)	16	6476	-945.67	1923.34	2031.75
Age (group), year	20	6476	-928.79	1897.58	2033.09
Age (cts), year	14	6476	-932.77	1893.54	1988.40
Age (cts, tvc), year	20	6476	-929.89	1899.77	2035.29
Type	14	6476	-967.40	1962.81	2057.67
Type, age (group)	24	6476	-941.83	1931.67	2094.29
Type, age (cts)	16	6476	-945.80	1923.61	2032.02
Type, age (cts, tvc)	22	6476	-942.71	1929.43	2078.50
Type, age (group), year	26	6476	-926.37	1904.74	2080.91
Type, age (cts), year	20	6476	-930.23	1900.46	2035.98
Type, age (cts, tvc), year	26	6476	-927.30	1906.60	2082.77
Age (group), year, management	24	6476	-927.82	1903.64	2066.26
Age (cts), year, management	18	6476	-931.82	1899.64	2021.60
Type, age (group), year, management	30	6476	-925.12	1910.24	2113.52
Type, age (cts), year, management	24	6476	-929.02	1906.05	2068.67
Age (group), year, calcification	24	6476	-927.63	1903.26	2065.88
Age (cts), year, calcification	18	6476	-931.60	1899.20	2021.17
Type, age (group), year, calcification	30	6476	-925.23	1910.46	2113.74
Type, age (cts), year, calcification	24	6476	-929.10	1906.20	2068.82
Age (spline)	14	6476	-945.80	1919.60	2014.46
Age (spline), year	18	6476	-930.17	1896.34	2018.31
Age (cts, linear, quadratic)	12	6476	-947.12	1918.24	1999.55
Age (cts, linear, quadratic), year	16	6476	-931.52	1895.03	2003.45
**Age (cts), year, density**	**18**	**6476**	**-927.37**	**1890.73**	**2012.70**
Age (cts), year, density, completeness	20	6476	-926.64	1893.27	2028.79

The AIC statistic show that the best model has age at diagnosis (as a continuous, linear variable), year of diagnosis and background parenchymal breast density as explanatory variables, whereas the BIC suggest that age at diagnosis alone (without year of diagnosis or background parenchymal

density) is the best model. We chose to use the age, year and background parenchymal density model since it is the best according to AIC, the descriptive statistics show that year of diagnosis was important, and clinical opinion that background parenchymal density is important. Adding type of atypia, management, and calcification to the age and year model did not improve the model fit statistics. Adding a variable of consecutive versus non-consecutive cases did not improve the model fit. Including age as a continuous, linear term was the best method, having better model fit statistics than the equivalent models for group, linear and quadratic and spline, showing the extra complexity to be unnecessary.

Table S14 Cancer rates (fitted values) at 1, 3, and 6 years since atypia diagnosis from main model (by age, year of diagnosis and background parenchymal breast density). Cumulative incidence of invasive cancer per 1000 women

Year of atypia diagnosis	Age	1 year		3 years		6 years	
		Est	95% CI	Est	95% CI	Est	95% CI
**High density**							
2003 to 2007	50	1.86	(0.14,3.59)	32.31	(18.75,45.86)	70.28	(44.75,95.8)
2003 to 2007	55	2.05	(0.17,3.93)	35.48	(21.41,49.54)	76.98	(51.05,102.9)
2003 to 2007	60	2.26	(0.18,4.33)	38.95	(23.14,54.76)	84.24	(55.01,113.46)
2003 to 2007	65	2.48	(0.15,4.81)	42.73	(23.69,61.77)	92.07	(56.18,127.97)
2003 to 2007	70	2.73	(0.08,5.38)	46.85	(23.05,70.66)	100.47	(54.7,146.25)
2008 to 2012	50	1.45	(0.12,2.77)	25.14	(14.6,35.68)	54.91	(35.25,74.56)
2008 to 2012	55	1.59	(0.14,3.04)	27.61	(16.44,38.79)	60.17	(39.65,80.68)
2008 to 2012	60	1.75	(0.14,3.36)	30.32	(17.51,43.12)	65.87	(42.12,89.61)
2008 to 2012	65	1.92	(0.11,3.74)	33.27	(17.69,48.85)	72	(42.4,101.61)
2008 to 2012	70	2.12	(0.05,4.19)	36.48	(16.95,56.01)	78.56	(40.66,116.45)
2013 to 2018	50	0.49	(0.02,0.96)	8.55	(4.13,12.97)	18.83	(9,28.67)
2013 to 2018	55	0.54	(0.03,1.05)	9.39	(4.63,14.15)	20.65	(10.09,31.21)
2013 to 2018	60	0.59	(0.02,1.16)	10.31	(4.93,15.7)	22.6	(10.7,34.5)
2013 to 2018	65	0.65	(0.01,1.29)	11.32	(4.95,17.68)	24.69	(10.73,38.64)
2013 to 2018	70	0.71	(0,1.44)*	12.4	(4.69,20.11)	26.87	(10.13,43.62)
**Low density**							
2003 to 2007	50	1.42	(0.09,2.74)	24.63	(13.85,35.41)	53.83	(33.19,74.48)
2003 to 2007	55	1.56	(0.13,2.99)	27.06	(16.32,37.8)	59.01	(39.1,78.92)
2003 to 2007	60	1.71	(0.15,3.28)	29.72	(18.2,41.23)	64.63	(43.53,85.72)
2003 to 2007	65	1.89	(0.15,3.62)	32.62	(19.19,46.04)	70.69	(45.71,95.67)
2003 to 2007	70	2.07	(0.11,4.04)	35.78	(19.19,52.37)	77.2	(45.54,108.86)
2008 to 2012	50	1.1	(0.09,2.11)	19.15	(10.99,27.31)	41.98	(26.63,57.32)
2008 to 2012	55	1.21	(0.11,2.3)	21.04	(12.78,29.3)	46.02	(30.97,61.07)
2008 to 2012	60	1.33	(0.13,2.53)	23.11	(14.04,32.17)	50.4	(33.96,66.85)
2008 to 2012	65	1.46	(0.12,2.81)	25.36	(14.59,36.13)	55.13	(35.11,75.14)
2008 to 2012	70	1.61	(0.08,3.13)	27.82	(14.38,41.25)	60.17	(34.45,85.88)
2013 to 2018	50	0.37	(0.02,0.73)	6.5	(3.14,9.85)	14.33	(6.85,21.8)
2013 to 2018	55	0.41	(0.02,0.79)	7.14	(3.64,10.64)	15.71	(7.92,23.5)
2013 to 2018	60	0.45	(0.02,0.87)	7.84	(3.99,11.69)	17.19	(8.66,25.73)

2013 to 2018	65	0.49	(0.02,0.97)	8.6	(4.13,13.07)	18.77	(8.95,28.6)
2013 to 2018	70	0.54	(0.01,1.08)	9.42	(4.05,14.79)	20.42	(8.72,32.12)
**Unrecorded density**							
2003 to 2007	50	1.84	(0,4.03)*	31.78	(4.64,58.92)	68.95	(12.78,125.13)
2003 to 2007	55	2.02	(0,4.41)*	34.87	(5.69,64.04)	75.35	(15.38,135.32)
2003 to 2007	60	2.22	(0,4.85)*	38.22	(6.3,70.14)	82.15	(17.02,147.29)
2003 to 2007	65	2.44	(0,5.36)*	41.84	(6.31,77.38)	89.29	(17.38,161.2)
2003 to 2007	70	2.69	(0,5.95)*	45.73	(5.58,85.87)	96.61	(16.2,177.02)
2008 to 2012	50	1.42	(0,3.14)*	24.71	(2.99,46.44)	53.78	(8.55,99)
2008 to 2012	55	1.57	(0,3.44)*	27.11	(3.62,50.6)	58.74	(10.15,107.32)
2008 to 2012	60	1.72	(0,3.79)*	29.71	(3.88,55.53)	63.98	(10.96,117)
2008 to 2012	65	1.89	(0,4.19)*	32.5	(3.67,61.33)	69.41	(10.73,128.1)
2008 to 2012	70	2.08	(0,4.66)*	35.48	(2.88,68.07)	74.86	(9.24,140.48)
2013 to 2018	50	0.48	(0,1.07)*	8.38	(0.62,16.15)	18.33	(1.36,35.3)
2013 to 2018	55	0.53	(0,1.17)*	9.19	(0.77,17.6)	19.96	(1.7,38.23)
2013 to 2018	60	0.58	(0,1.29)*	10.05	(0.81,19.29)	21.64	(1.77,41.52)
2013 to 2018	65	0.64	(0,1.43)*	10.96	(0.68,21.25)	23.29	(1.48,45.1)
2013 to 2018	70	0.7	(0,1.59)*	11.9	(0.35,23.46)	24.79	(0.77,48.8)

***The assumptions used to calculate the confidence intervals can occasionally lead to the lower bound taking a small negative value. These are given as zero in the table.**

The CIF for the causes in the main model (invasive cancer and death) are evaluated at 1, 3 and 6 years in table S14. For someone aged 60 with high background parenchymal density the estimated rate of invasive cancer at 3 years for those diagnosed with atypia between 2003 and 2007 was 38.95 per 1000 women, 95% CI (23.14,49.54), and for those diagnosed with atypia between 2013 and 2018 was 10.31 per 1000 women, 95% CI (4.93,15.70). For low background parenchymal density, the corresponding rates were 29.72, (18.20,41.23), and 7.84, (3.99,11.69).

Table S15 Comparison of the risk of subsequent invasive cancers considering different factors

**Comparison**	**Hazard ratio**	**95% CI**	**p**
**Main model**			
Age*	1.019	0.996, 1.043	0.110
Low background parenchymal breast density vs high	0.760	0.537, 1.075	0.120
Years 2008 to 2013 vs years 2003 to 2007	0.775	0.525, 1.145	0.201
Years 2013 to 2018 vs years 2003 to 2007	0.262	0.149, 0.461	<0.001
**Individual models with variables added to main model in turn**			
Variable: Atypia type			
FEA vs ADH	1.167	0.546, 2.494	0.690
LISN vs ADH	1.137	0.777, 1.663	0.509
Mixed vs ADH	1.712	1.054, 2.782	0.030
Variable: Management			
Single diagnostic needle biopsy vs Second line vacuum assisted biopsy/excision	0.771	0.368, 1.618	0.492
Single diagnostic needle biopsy vs Surgery	0.750	0.451, 1.246	0.267

Surgery vs Second line vacuum assisted biopsy/excision	1.029	0.543, 1.948	0.930
Variable: consecutive cases			
Non-consecutive vs consecutive cases	1.010	0.705, 1.447	0.957

*Age is a continuous variable measured in years, so the change is over a period of one year

The hazard ratios presented in Table S15 come from different models. Age, density and year of diagnosis was compared in the main model. Other variables were added in turn to the main model to derive hazard ratios for the relevant comparisons.

Figure S6 Cause-specific hazard function for each cause of outcome from the main model by time since atypia diagnosis. Shown by age at diagnosis, year of diagnosis and background parenchymal density (high in figure a and low in figure b)

a<br>High<br>density	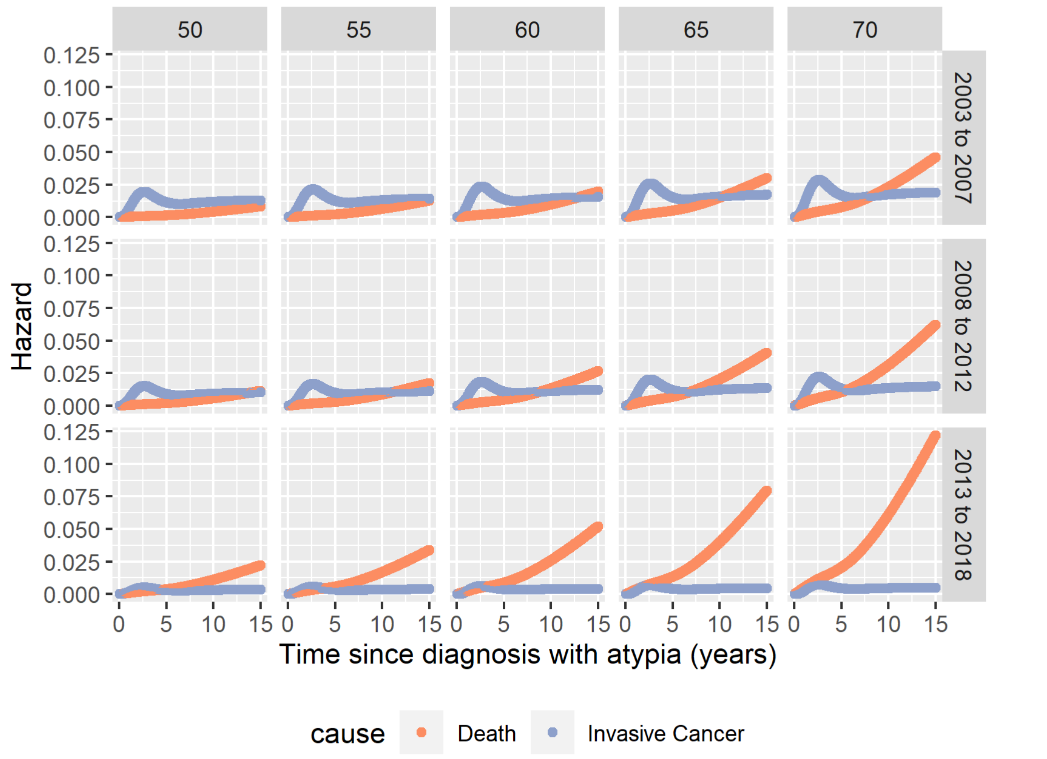
b<br>Low<br>density	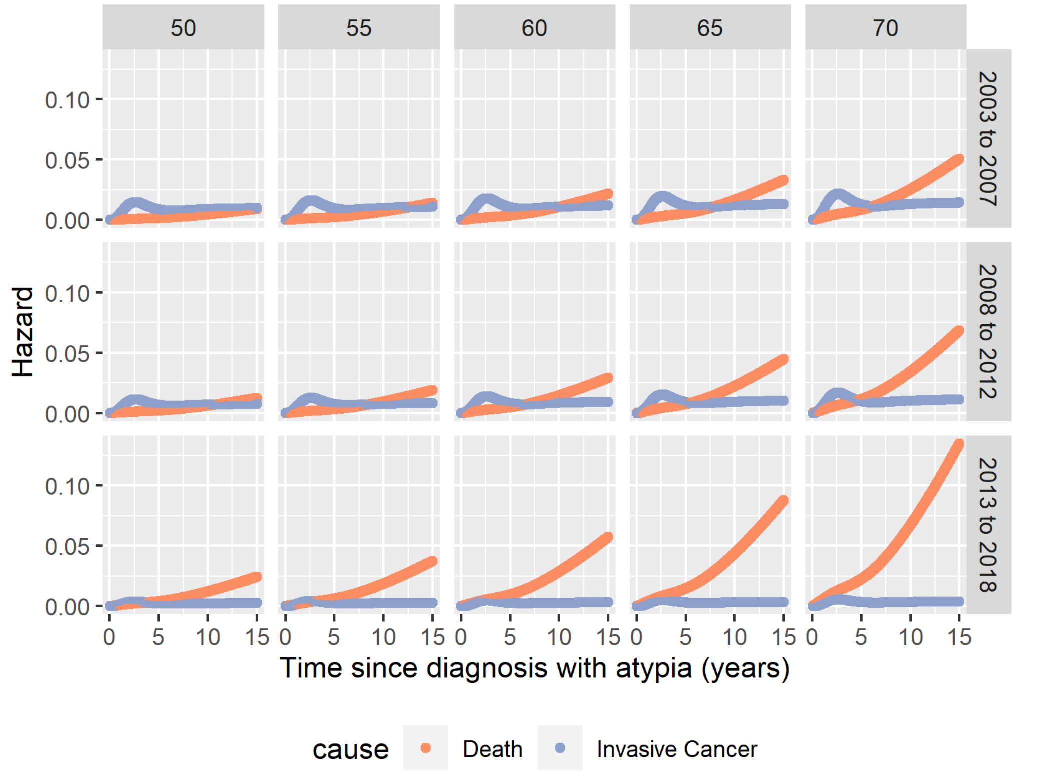

Figure S7 Stacked cumulative incidence plots from the main model. Show cumulative incidence of death and invasive cancer since diagnosis with atypia for people aged 50, 55, 60, 65, and 70 at time of diagnosis and diagnosed in the three periods: 2003 to 2007, 2008 to 2012, and 2013 to 2018; with high background parenchymal density shown in figure a and low background parenchymal density in figure b

a
High density

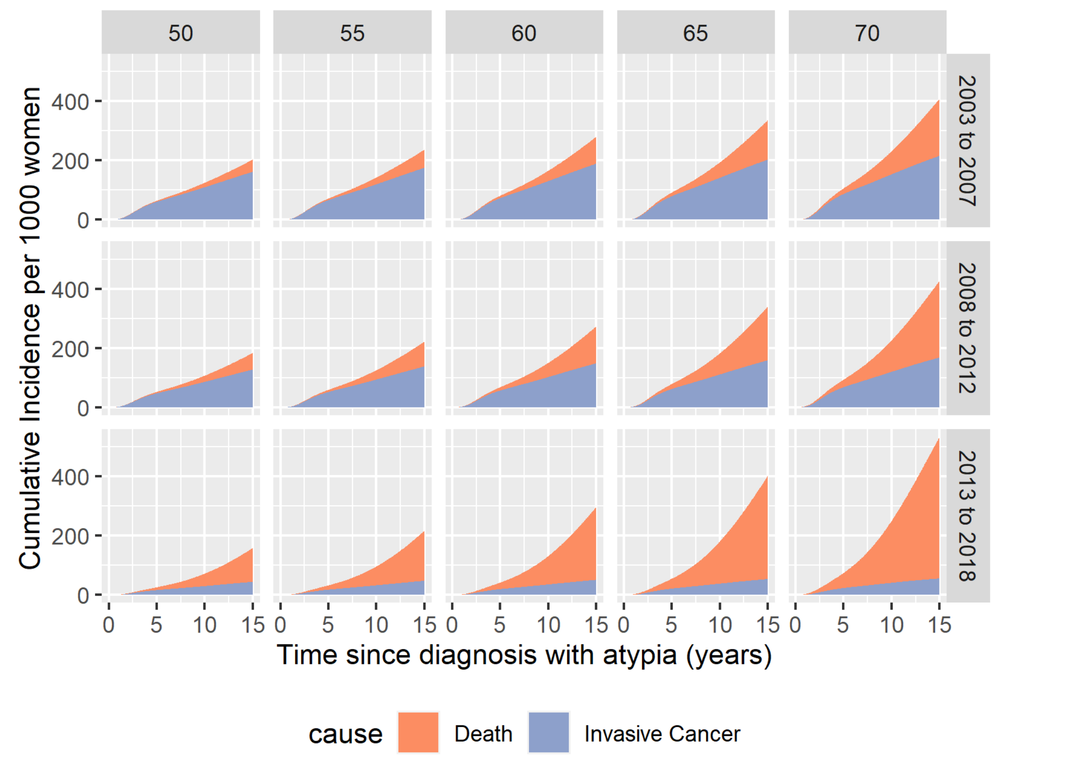

b
Low density

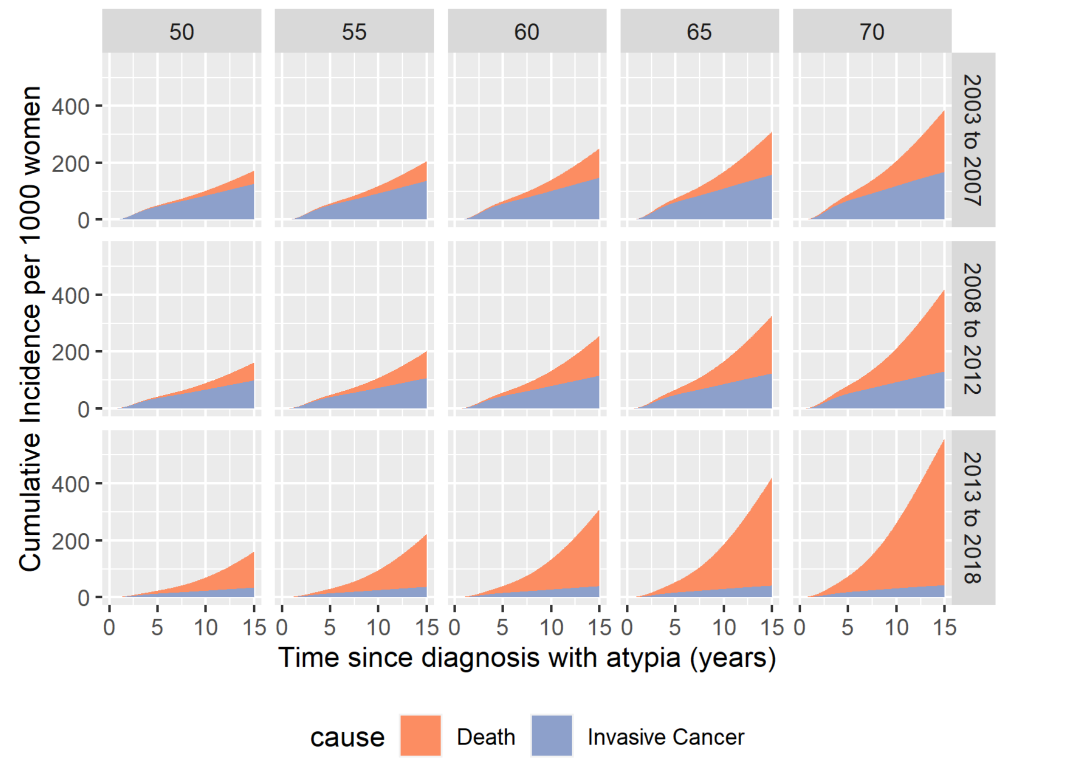

Results for invasive cancer split into ipsilateral and contralateral cancers

Figure S8 Cause-specific hazards (a, b), stacked cumulative incidence functions (c, d) and cumulative incidence functions (e, f) evaluated at 1 year, 3 years, and 6 years since diagnosis with atypia, from the main model with death and invasive cancer split into ipsilateral and contralateral. Only ipsilateral and contralateral cancers are shown.

a
High density

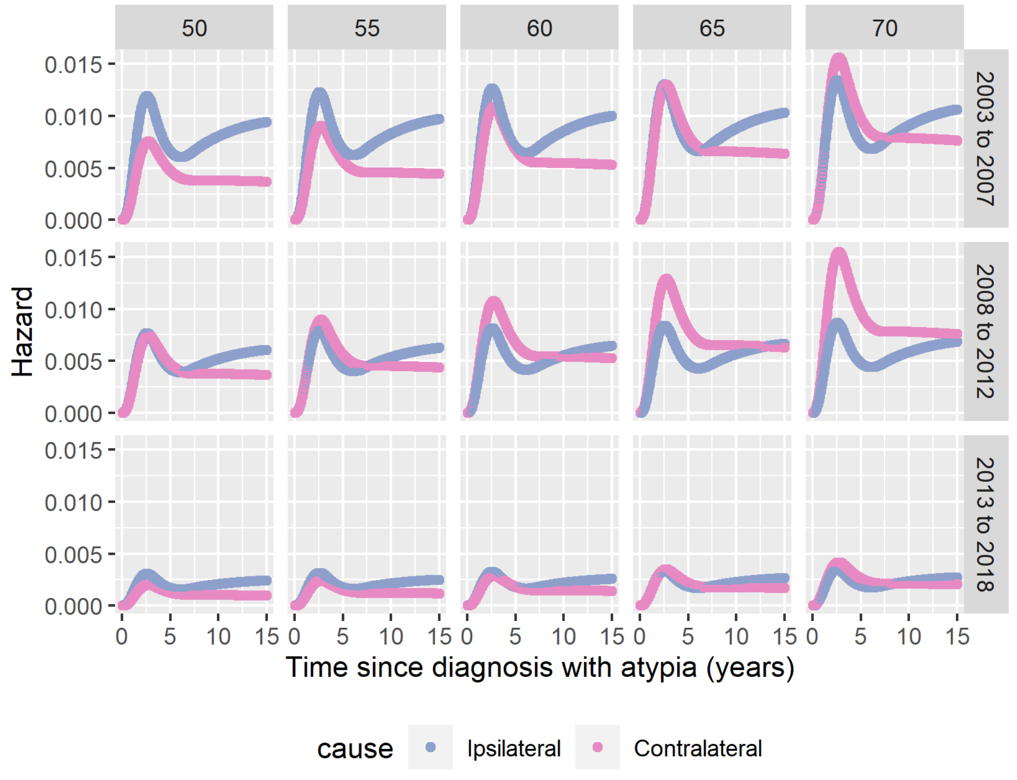

b
Low density

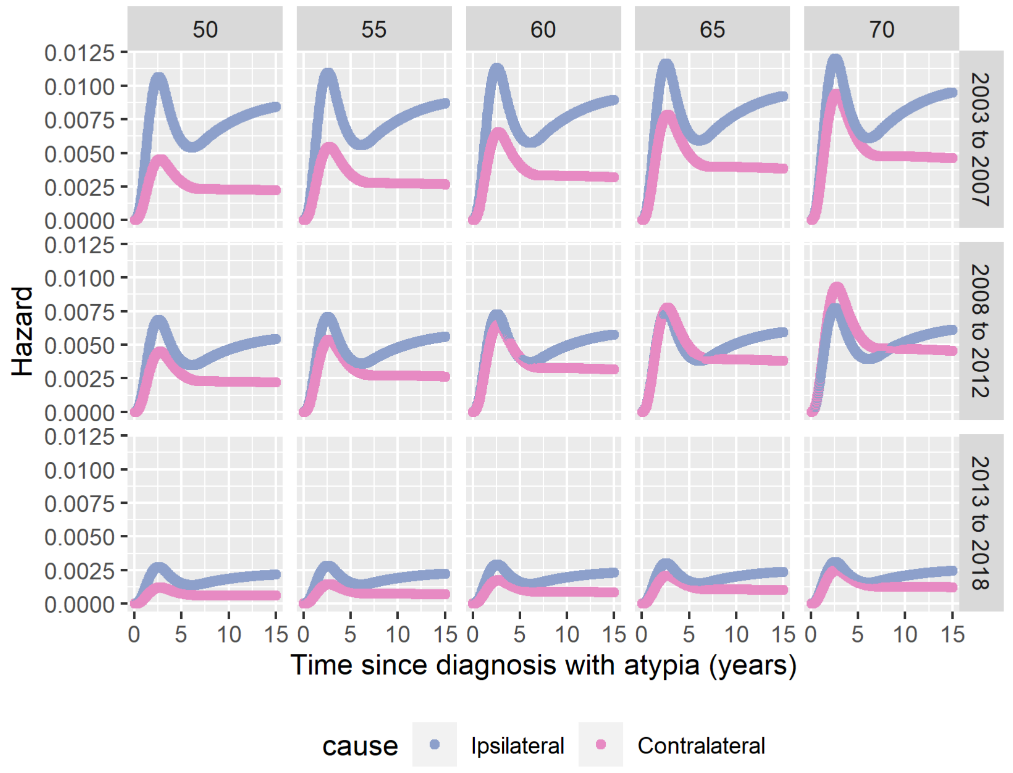

c<br>High density	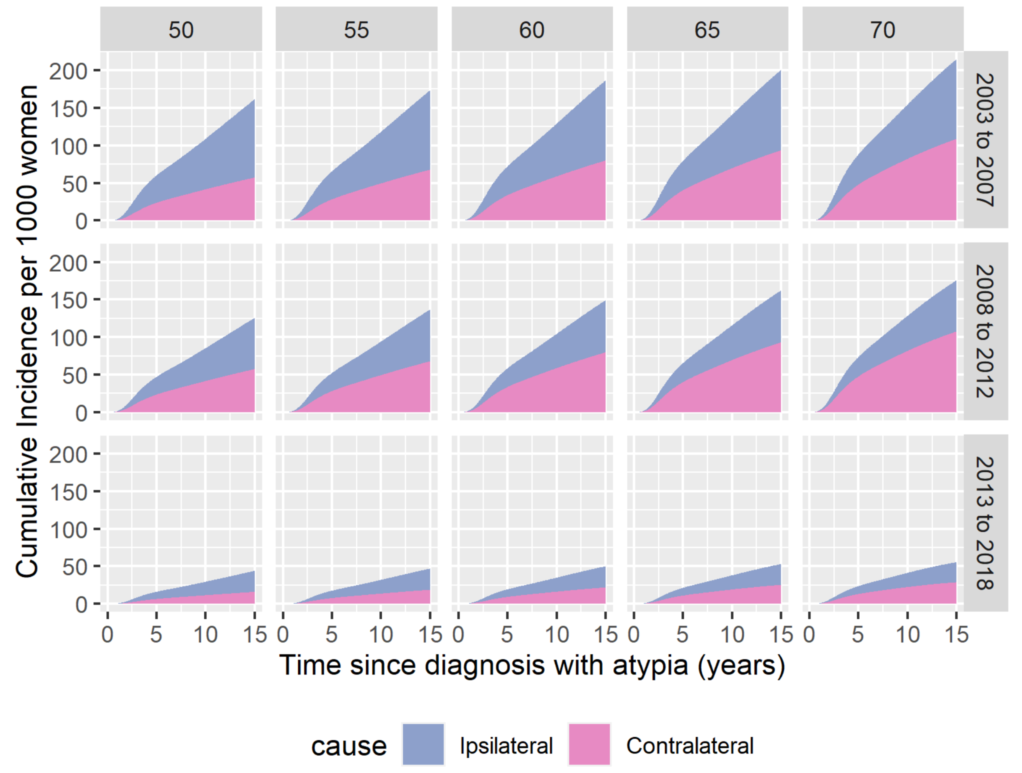
d<br>Low density	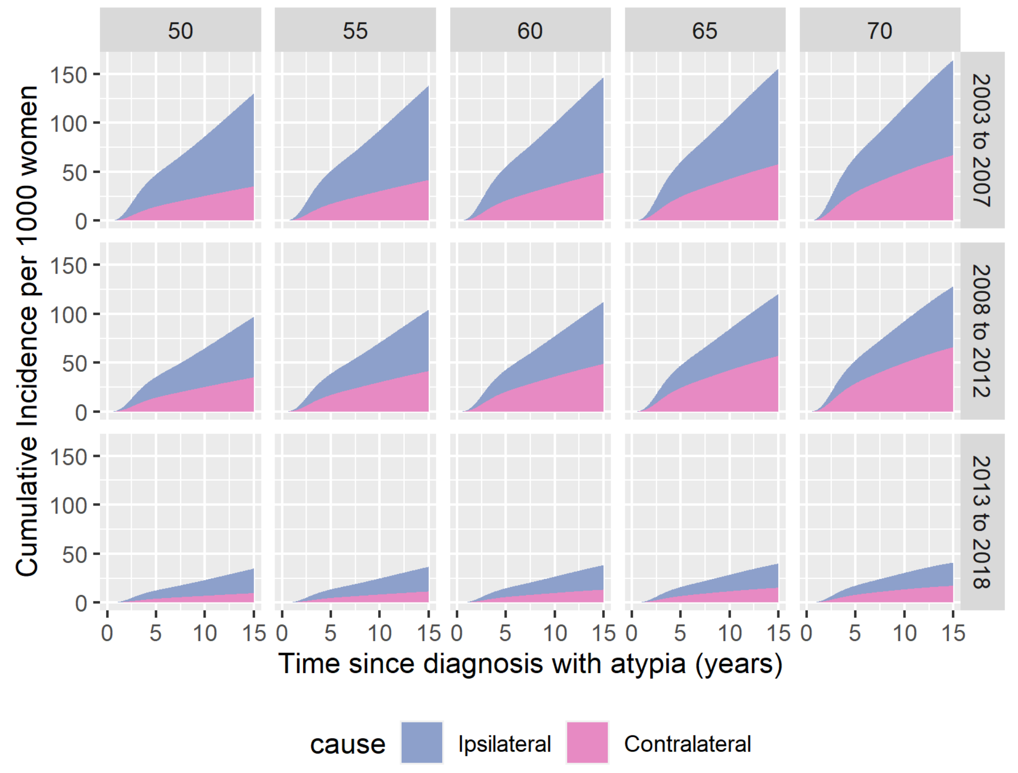

e
High
density

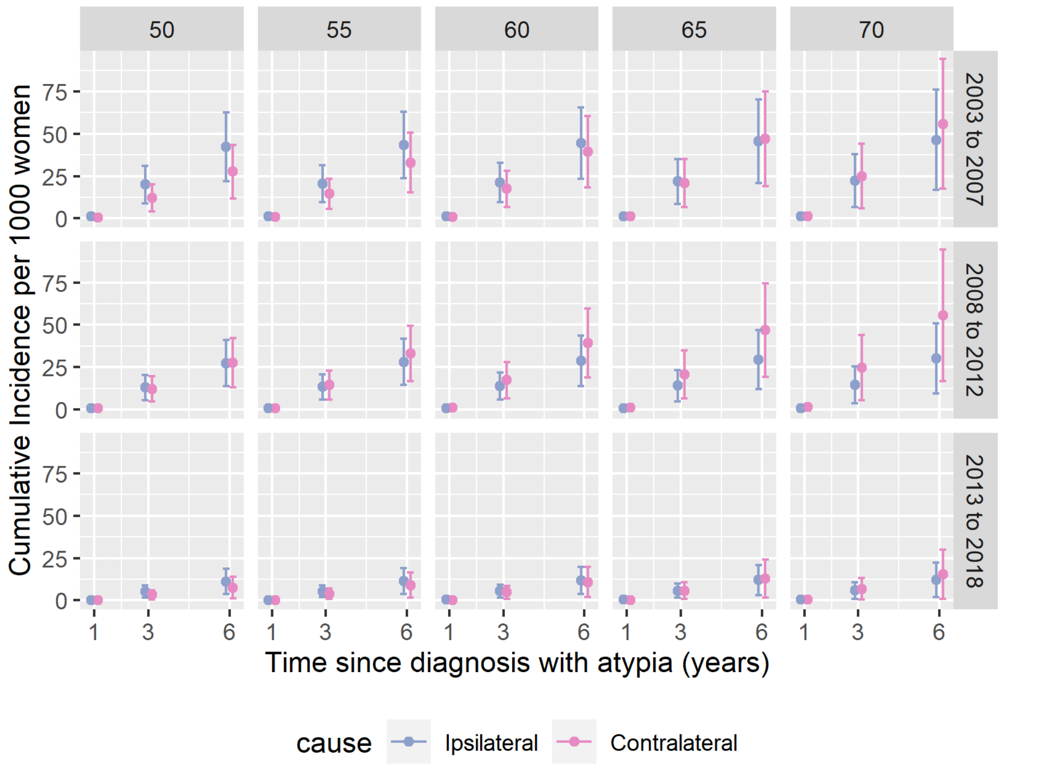

f
Low
density

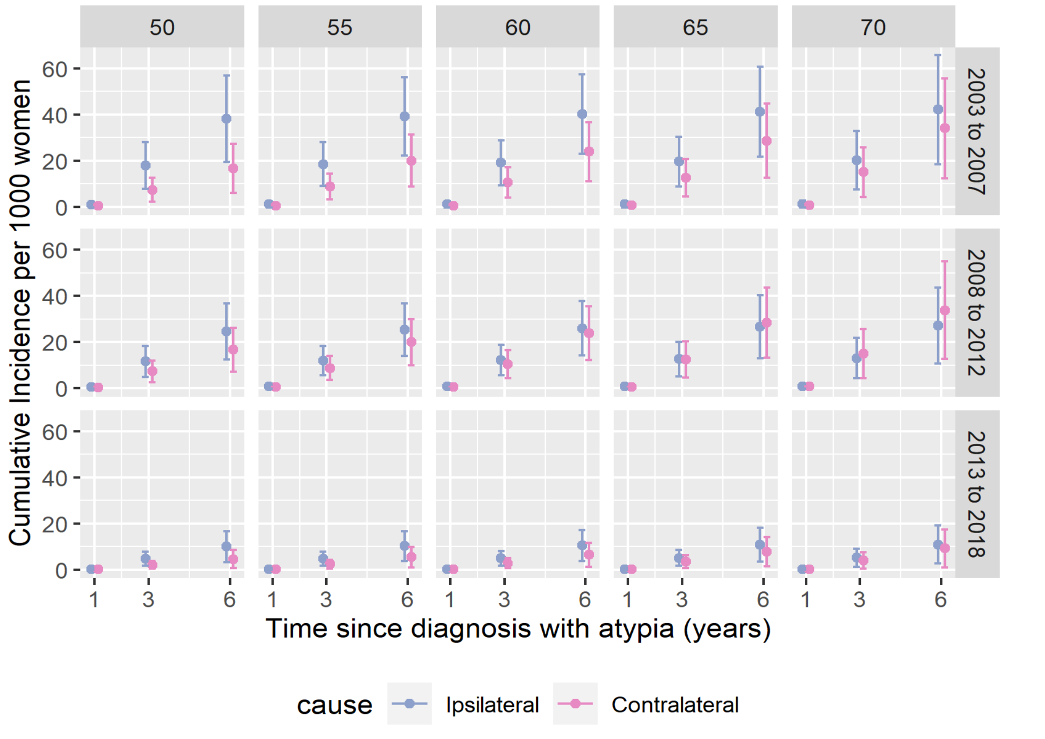

Model when the outcome is the earlier of invasive cancer or DCIS

Table S16 Fitted values at 1, 3, and 6 years since atypia diagnosis from main model with invasive cancer and DCIS combined. Cumulative incidence of outcome cause per 1000 women.

		1 year		3 years		6 years	
Year	Age	Est.	95% CI	Est.	95% CI	Est.	95% CI
High density							
2003 to 2007	50	1.21	(0.07,2.36)	25.3	(14.58,36.02)	62.34	(39.94,84.75)
2003 to 2007	55	1.33	(0.09,2.58)	27.72	(16.49,38.95)	68.12	(45.12,91.13)
2003 to 2007	60	1.46	(0.09,2.83)	30.36	(17.81,42.91)	74.37	(48.59,100.15)
2003 to 2007	65	1.6	(0.07,3.13)	33.23	(18.38,48.08)	81.1	(50,112.2)
2003 to 2007	70	1.76	(0.03,3.48)	36.35	(18.17,54.53)	88.28	(49.37,127.19)
2008 to 2012	50	1.1	(0.08,2.12)	22.92	(13.54,32.3)	56.55	(37.48,75.63)
2008 to 2012	55	1.2	(0.09,2.32)	25.11	(15.14,35.08)	61.79	(41.86,81.72)
2008 to 2012	60	1.32	(0.09,2.55)	27.5	(16.16,38.83)	67.44	(44.51,90.37)
2008 to 2012	65	1.45	(0.07,2.82)	30.09	(16.5,43.69)	73.5	(45.22,101.78)
2008 to 2012	70	1.59	(0.03,3.15)	32.91	(16.12,49.7)	79.93	(44.1,115.77)
2013 to 2018	50	0.64	(0.04,1.23)	13.34	(7.75,18.92)	33.09	(19.08,47.1)
2013 to 2018	55	0.7	(0.05,1.35)	14.61	(8.65,20.58)	36.15	(21.23,51.06)
2013 to 2018	60	0.77	(0.05,1.48)	15.99	(9.2,22.79)	39.42	(22.54,56.29)
2013 to 2018	65	0.84	(0.04,1.64)	17.49	(9.36,25.63)	42.88	(22.87,62.88)
2013 to 2018	70	0.92	(0.02,1.83)	19.11	(9.09,29.12)	46.46	(22.16,70.77)
Low density							
2003 to 2007	50	0.95	(0.05,1.84)	19.77	(11.12,28.41)	48.9	(30.57,67.23)
2003 to 2007	55	1.04	(0.07,2.01)	21.66	(12.92,30.4)	53.46	(35.5,71.42)
2003 to 2007	60	1.14	(0.08,2.2)	23.72	(14.33,33.12)	58.4	(39.3,77.49)
2003 to 2007	65	1.25	(0.08,2.42)	25.97	(15.16,36.79)	63.71	(41.43,85.99)
2003 to 2007	70	1.37	(0.06,2.68)	28.42	(15.35,41.49)	69.39	(41.74,97.03)
2008 to 2012	50	0.86	(0.06,1.65)	17.9	(10.49,25.31)	44.32	(29.14,59.5)
2008 to 2012	55	0.94	(0.08,1.8)	19.61	(12.06,27.17)	48.45	(33.51,63.38)
2008 to 2012	60	1.03	(0.08,1.97)	21.48	(13.22,29.74)	52.9	(36.64,69.16)
2008 to 2012	65	1.13	(0.08,2.18)	23.51	(13.82,33.2)	57.68	(38.08,77.27)
2008 to 2012	70	1.24	(0.06,2.42)	25.72	(13.82,37.61)	62.74	(37.84,87.64)
2013 to 2018	50	0.5	(0.03,0.96)	10.4	(6.07,14.74)	25.86	(14.96,36.76)
2013 to 2018	55	0.54	(0.04,1.04)	11.4	(6.95,15.84)	28.25	(17.09,39.4)
2013 to 2018	60	0.6	(0.05,1.14)	12.48	(7.6,17.35)	30.8	(18.62,42.98)
2013 to 2018	65	0.65	(0.05,1.26)	13.64	(7.92,19.37)	33.49	(19.33,47.66)
2013 to 2018	70	0.72	(0.03,1.4)	14.9	(7.88,21.92)	36.27	(19.15,53.4)
Unrecorded density							
2003 to 2007	50	1.39	(0,2.99)*	28.95	(6.78,51.13)	70.91	(19.88,121.93)
2003 to 2007	55	1.53	(0,3.26)*	31.68	(7.92,55.45)	77.24	(23,131.48)
2003 to 2007	60	1.68	(0,3.58)*	34.64	(8.7,60.58)	83.94	(25.21,142.67)
2003 to 2007	65	1.84	(0,3.94)*	37.82	(9,66.65)	90.92	(26.22,155.63)
2003 to 2007	70	2.02	(0,4.36)*	41.22	(8.7,73.75)	98	(25.74,170.25)
2008 to 2012	50	1.26	(0,2.71)*	26.22	(5.74,46.69)	64.24	(17.1,111.38)

2008 to 2012	55	1.38	(0,2.96)*	28.68	(6.62,50.74)	69.91	(19.53,120.28)
2008 to 2012	60	1.52	(0,3.25)*	31.34	(7.16,55.51)	75.85	(21.1,130.61)
2008 to 2012	65	1.66	(0,3.58)*	34.18	(7.24,61.12)	81.95	(21.51,142.38)
2008 to 2012	70	1.82	(0,3.97)*	37.2	(6.78,67.62)	87.96	(20.54,155.39)
2013 to 2018	50	0.73	(0,1.57)*	15.23	(3.34,27.12)	37.41	(8.36,66.46)
2013 to 2018	55	0.8	(0,1.71)*	16.64	(3.83,29.45)	40.57	(9.53,71.61)
2013 to 2018	60	0.88	(0,1.88)*	18.14	(4.1,32.18)	43.76	(10.15,77.36)
2013 to 2018	65	0.96	(0,2.07)*	19.72	(4.11,35.33)	46.82	(10.07,83.57)
2013 to 2018	70	1.05	(0,2.29)*	21.34	(3.78,38.9)	49.5	(9.11,89.9)

*The assumptions used to calculate the confidence intervals can occasionally lead to the lower bound taking a small negative value. These are given as zero in the table

Figure S9 Cumulative incidence functions evaluated at 1 year, 3 years, and 6 years since diagnosis with atypia, from the main model with invasive cancer or DCIS combined for high (a) and low (b) background parenchymal density

a
High density

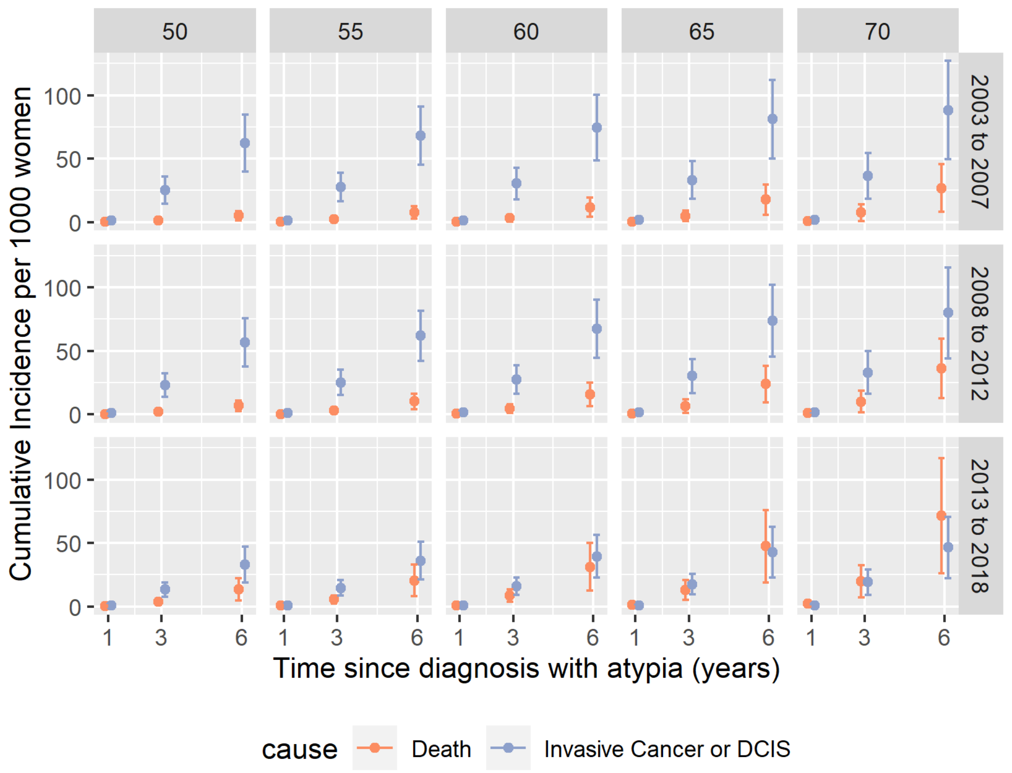

b
Low density

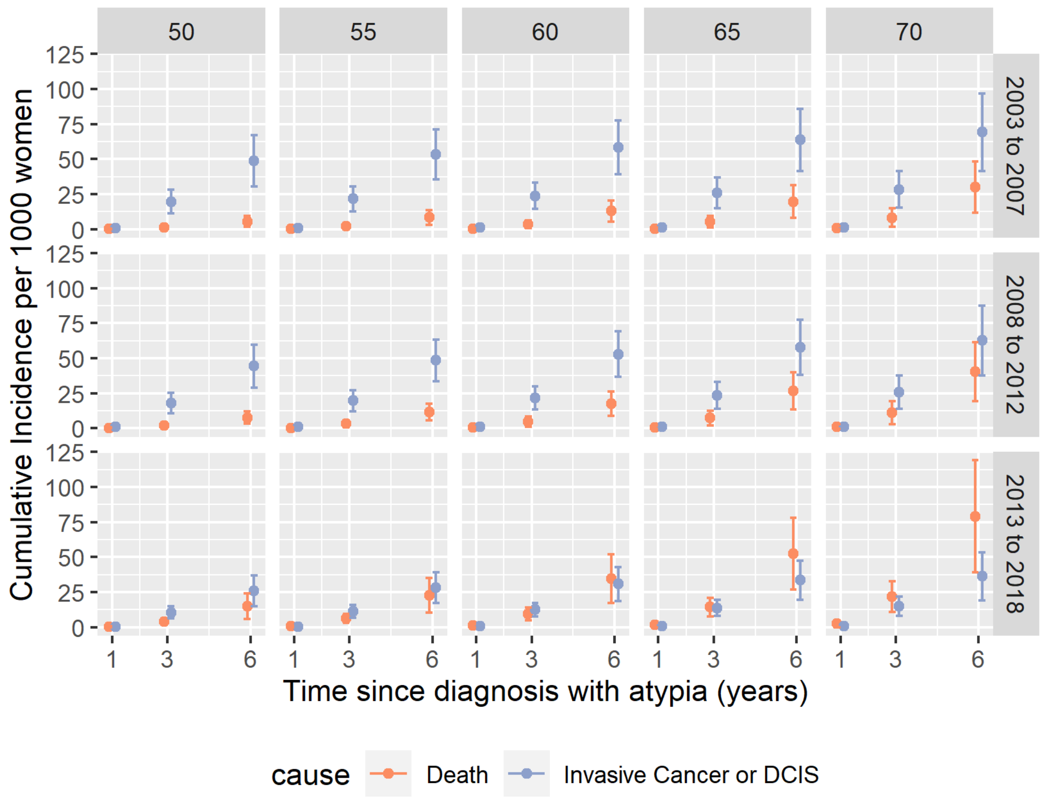

3.2 Modelling of cancer rates using the subdistribution method

Table S17 Cancer rates (fitted values) at 1, 3, and 6 years since atypia diagnosis from main model, using the subdistribution method. Cumulative incidence of invasive cancer per 1000 women

		1 year		3 years		6 years	
Year	Age	Est	95% CI	Est	95% CI	Est	95% CI
**Invasive cancer, high density**							
2003 to 2007	50	2.03	(0.62,6.62)	28.47	(18,44.88)	74.65	(51.55,107.51)
2003 to 2007	55	2.21	(0.68,7.14)	30.96	(20.08,47.6)	81.02	(57.68,113.21)
2003 to 2007	60	2.4	(0.74,7.78)	33.67	(21.81,51.82)	87.9	(62.46,123)
2003 to 2007	65	2.62	(0.8,8.56)	36.61	(23.05,57.91)	95.34	(65.53,137.68)
2003 to 2007	70	2.85	(0.85,9.52)	39.81	(23.84,66.1)	103.37	(67.09,157.52)
2008 to 2012	50	1.55	(0.47,5.05)	21.76	(13.73,34.39)	57.38	(39.56,82.86)
2008 to 2012	55	1.68	(0.52,5.46)	23.67	(15.22,36.72)	62.32	(43.95,88.01)
2008 to 2012	60	1.83	(0.56,5.96)	25.75	(16.42,40.26)	67.67	(47.25,96.47)
2008 to 2012	65	1.99	(0.6,6.58)	28.01	(17.28,45.25)	73.47	(49.3,108.79)
2008 to 2012	70	2.17	(0.64,7.33)	30.46	(17.8,51.89)	79.74	(50.31,125.2)
2013 to 2018	50	0.52	(0.15,1.71)	7.3	(4.33,12.29)	19.5	(11.78,32.19)
2013 to 2018	55	0.56	(0.17,1.85)	7.95	(4.78,13.2)	21.21	(12.99,34.54)
2013 to 2018	60	0.61	(0.18,2.03)	8.66	(5.15,14.52)	23.08	(13.99,37.97)
2013 to 2018	65	0.67	(0.2,2.24)	9.42	(5.43,16.32)	25.11	(14.72,42.68)
2013 to 2018	70	0.72	(0.21,2.5)	10.26	(5.62,18.69)	27.31	(15.19,48.85)
**Invasive cancer, low density**							
2003 to 2007	50	1.54	(0.48,5)	21.75	(14.04,33.62)	57.35	(40.29,81.33)
2003 to 2007	55	1.68	(0.52,5.38)	23.66	(15.75,35.47)	62.29	(45.43,85.13)
2003 to 2007	60	1.83	(0.57,5.86)	25.74	(17.17,38.49)	67.64	(49.46,92.17)
2003 to 2007	65	1.99	(0.62,6.44)	28	(18.19,42.97)	73.44	(51.99,103.24)
2003 to 2007	70	2.17	(0.66,7.15)	30.45	(18.81,49.1)	79.7	(53.2,118.56)
2008 to 2012	50	1.18	(0.36,3.81)	16.61	(10.73,25.65)	43.98	(30.99,62.25)
2008 to 2012	55	1.28	(0.4,4.11)	18.07	(11.96,27.26)	47.8	(34.67,65.73)
2008 to 2012	60	1.39	(0.43,4.48)	19.66	(12.95,29.8)	51.94	(37.44,71.85)
2008 to 2012	65	1.52	(0.47,4.94)	21.39	(13.64,33.47)	56.43	(39.1,81.12)
2008 to 2012	70	1.65	(0.5,5.5)	23.28	(14.05,38.43)	61.3	(39.86,93.69)
2013 to 2018	50	0.39	(0.12,1.32)	5.56	(3.22,9.59)	14.88	(8.73,25.3)
2013 to 2018	55	0.43	(0.13,1.42)	6.06	(3.57,10.28)	16.19	(9.65,27.09)
2013 to 2018	60	0.47	(0.14,1.55)	6.6	(3.86,11.26)	17.62	(10.43,29.68)
2013 to 2018	65	0.51	(0.15,1.71)	7.18	(4.08,12.62)	19.17	(11.03,33.23)
2013 to 2018	70	0.55	(0.16,1.91)	7.82	(4.24,14.4)	20.86	(11.43,37.9)
**Invasive cancer, unrecorded density**							
2003 to 2007	50	1.93	(0.47,7.93)	27.1	(11.1,65.4)	71.15	(30.78,159.9)
2003 to 2007	55	2.1	(0.51,8.55)	29.48	(12.25,70.07)	77.22	(33.94,170.59)
2003 to 2007	60	2.29	(0.56,9.31)	32.06	(13.33,76.06)	83.8	(36.91,184.29)
2003 to 2007	65	2.49	(0.61,10.22)	34.86	(14.31,83.62)	90.91	(39.56,201.53)
2003 to 2007	70	2.71	(0.65,11.31)	37.9	(15.17,93.05)	98.58	(41.83,222.83)
2008 to 2012	50	1.47	(0.35,6.08)	20.71	(8.38,50.72)	54.66	(23.3,125.41)

2008 to 2012	55	1.6	(0.39,6.58)	22.53	(9.21,54.55)	59.37	(25.62,134.44)
2008 to 2012	60	1.74	(0.42,7.18)	24.51	(10,59.44)	64.48	(27.77,145.96)
2008 to 2012	65	1.9	(0.46,7.9)	26.66	(10.71,65.6)	70.02	(29.68,160.43)
2008 to 2012	70	2.07	(0.49,8.76)	29	(11.32,73.26)	76	(31.32,178.3)
2013 to 2018	50	0.49	(0.12,2.01)	6.95	(2.81,17.16)	18.56	(7.61,44.9)
2013 to 2018	55	0.53	(0.13,2.18)	7.56	(3.08,18.51)	20.19	(8.35,48.39)
2013 to 2018	60	0.58	(0.14,2.38)	8.23	(3.34,20.23)	21.97	(9.05,52.84)
2013 to 2018	65	0.63	(0.15,2.62)	8.96	(3.57,22.41)	23.9	(9.67,58.45)
2013 to 2018	70	0.69	(0.16,2.91)	9.76	(3.77,25.13)	26	(10.2,65.44)

Figure S10 Cumulative incidence function for invasive cancer from the main model using the subdistribution method, evaluated at 1 year, 3 years, and 6 years since diagnosis with atypia; by age at diagnosis, year of diagnosis and background parenchymal density (high in figure a, low in figure b)

a
High density

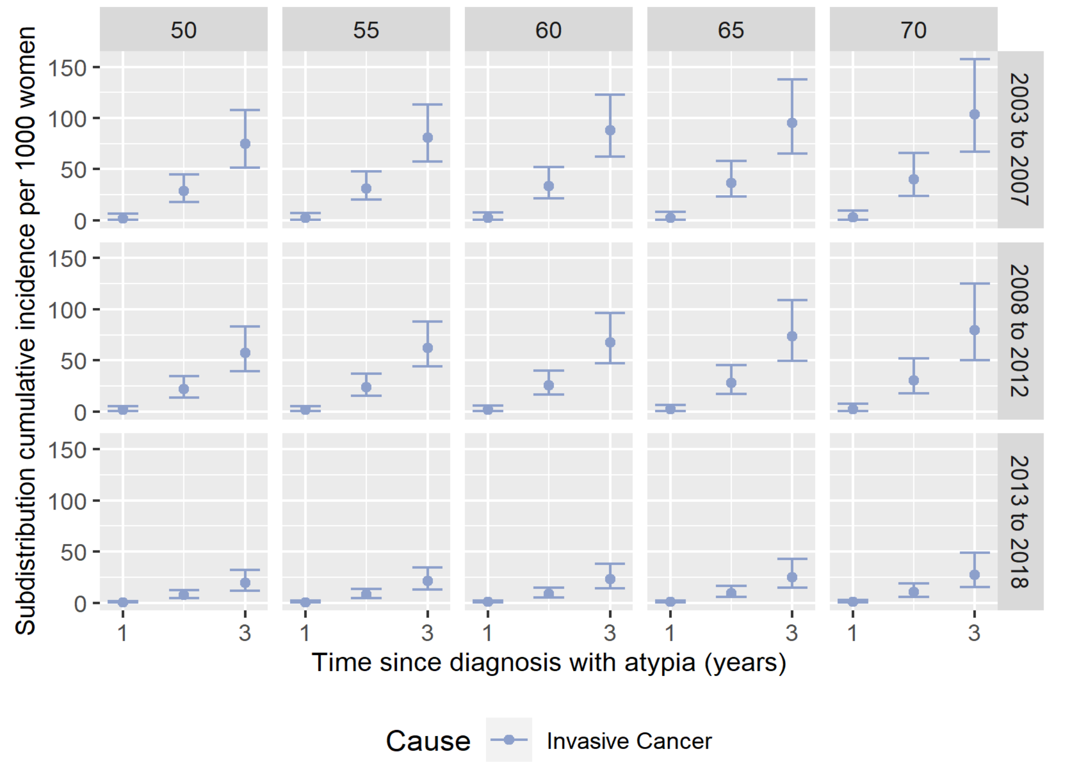

b
Low density

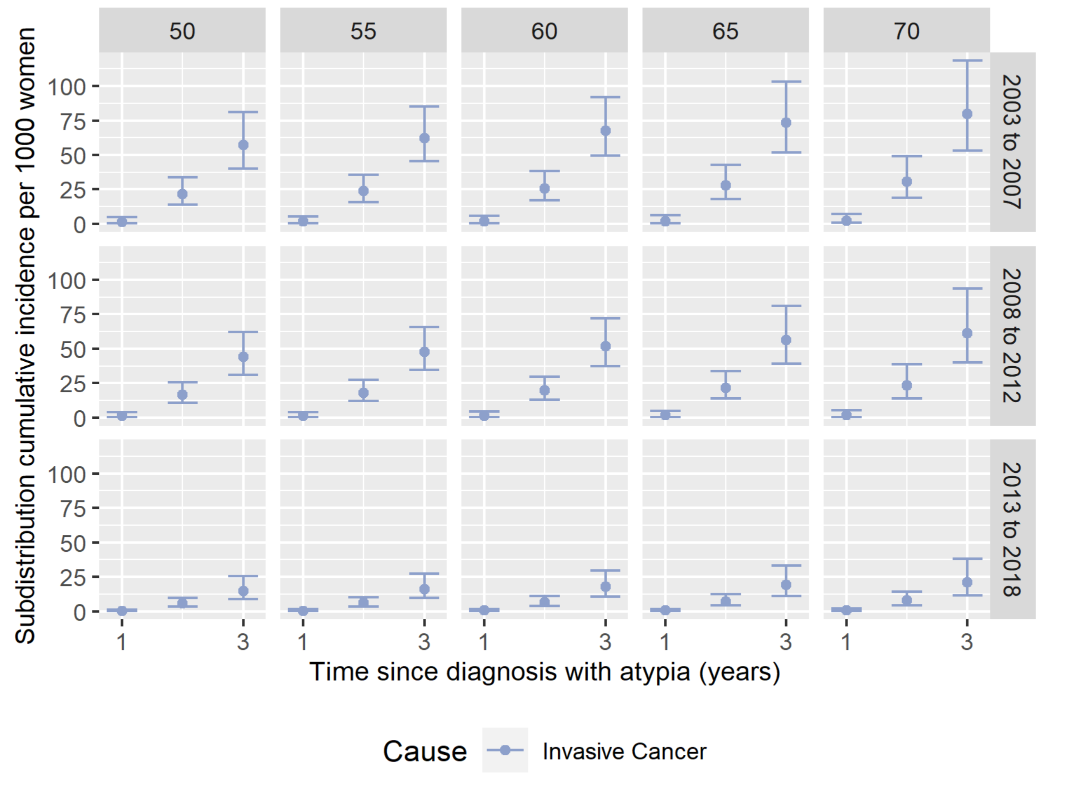

Supplement: Supplementary file 1 — Web appendix: Supplementary material [file frek077039.ww.pdf]
